# LncRNA *GACAT2* binds with protein PKM1/2 to regulate cell mitochondrial function and cementogenesis in an inflammatory environment

**DOI:** 10.1038/s41413-022-00197-x

**Published:** 2022-03-16

**Authors:** Xuan Li, Bei-Min Tian, Dao-Kun Deng, Fen Liu, Huan Zhou, De-Qin Kong, Hong-Lei Qu, Li-Juan Sun, Xiao-Tao He, Fa-Ming Chen

**Affiliations:** 1 State Key Laboratory of Military Stomatology & National Clinical Research Center for Oral Diseases & Shaanxi Engineering Research Center for Dental Materials and Advanced Manufacture, Department of Periodontology, School of Stomatology, The Fourth Military Medical University, Xi’an, 710032 P. R. China; 2 Department of Stomatology, Northwest Women’s and Children’s Hospital, Xi’an, 710032 P. R. China; 3 Key Laboratory of Shaanxi Province for Craniofacial Precision Medicine Research, Department of Periodontology, College of Stomatology, Xi’an Jiaotong University, Xi’an, 710032 P. R. China; 4 Department of Toxicology, Shaanxi Provincial Key Laboratory of Free Radical Biology and Medicine, The Ministry of Education Key Laboratory of Hazard Assessment and Control in Special Operational Environment, School of Public Health, Fourth Military Medical University, Xi’an, 710032 P. R. China

**Keywords:** Energy metabolism, Bone, Pathogenesis

## Abstract

Periodontal ligament stem cells (PDLSCs) are a key cell type for restoring/regenerating lost/damaged periodontal tissues, including alveolar bone, periodontal ligament and root cementum, the latter of which is important for regaining tooth function. However, PDLSCs residing in an inflammatory environment generally exhibit compromised functions, as demonstrated by an impaired ability to differentiate into cementoblasts, which are responsible for regrowing the cementum. This study investigated the role of mitochondrial function and downstream long noncoding RNAs (lncRNAs) in regulating inflammation-induced changes in the cementogenesis of PDLSCs. We found that the inflammatory cytokine-induced impairment of the cementogenesis of PDLSCs was closely correlated with their mitochondrial function, and lncRNA microarray analysis and gain/loss-of-function studies identified *GACAT2* as a regulator of the cellular events involved in inflammation-mediated mitochondrial function and cementogenesis. Subsequently, a comprehensive identification of RNA-binding proteins by mass spectrometry (ChIRP-MS) and parallel reaction monitoring (PRM) assays revealed that *GACAT2* could directly bind to pyruvate kinase M1/2 (PKM1/2), a protein correlated with mitochondrial function. Further functional studies demonstrated that *GACAT2* overexpression increased the cellular protein expression of PKM1/2, the PKM2 tetramer and phosphorylated PKM2, which led to enhanced pyruvate kinase (PK) activity and increased translocation of PKM2 into mitochondria. We then found that *GACAT2* overexpression could reverse the damage to mitochondrial function and cementoblastic differentiation of PDLSCs induced by inflammation and that this effect could be abolished by PKM1/2 knockdown. Our data indicated that by binding to PKM1/2 proteins, the lncRNA *GACAT2* plays a critical role in regulating mitochondrial function and cementogenesis in an inflammatory environment.

## Introduction

Periodontitis is a chronic inflammatory disease that causes progressive destruction of the periodontium, a highly hierarchically organized organ that includes alveolar bone, periodontal ligament (PDL) and root cementum, and if left untreated, this disease causes tooth movement and eventually leads to tooth loss.^1,2^ In support of combating tissue loss, techniques ranging from guided tissue regeneration (GTR) to bone grafting and, more recently, stem cell-based regenerative therapies have either been used or paved the way for the current protocols for periodontal treatment.^3–5^ Unfortunately, these therapeutic paradigms are either classified as crude “regeneration-promoting” methods or have poor clinical predictability. The successful regeneration of lost/damaged periodontal tissues remains a challenge due to the difficulty of tissue regrowth within an inflammatory environment and the need to reconstruct at least three types of tissues as an integrated, functional unit.^3–6^ As a fundamental component of the tooth-supporting apparatus, the cementum is responsible for connecting the PDL to the tooth and thus plays a vitally important role in restoring tooth function; however, the regeneration of damaged cementum on a diseased root surface has not been successfully achieved in the field of regenerative medicine.^6–8^

The postnatal PDL constitutes an important stem cell source, *i.e*., PDL stem cells (PDLSCs) that have been proven to differentiate into osteoblasts, fibroblasts and cementoblasts under appropriate conditions, wherein cementoblasts are the progenitor responsible for cementum regeneration and the specialized cell type that can form mineralized cementum tissue.^9,10^ Although PDLSCs can give rise to osteoblastic and cementoblastic lineages and represent the optimal candidate for periodontal regeneration, substantial evidence suggests that PDLSCs within a diseased periodontium exhibit a compromised function and are thus unable to properly differentiate into the required cell types to form correct periodontal tissues.^9,11–15^ By targeting the osteogenesis of PDLSCs, we identified the P2X7 receptor (P2X7R) as a key molecule that can be used to reverse the inflammation-compromised osteogenesis of PDLSCs.^16^ Via a paracrine mechanism, the overexpression of P2X7R allowed PDLSCs to exert positive effects on their neighboring cells, which protected them against inflammation and thus ensured appropriate osteogenic differentiation.^13^ In regard to the cementogenesis of PDLSCs, emerging evidence has indicated that inflammatory cytokines, including TNF-α and IL-1β, inhibit cementoblastic differentiation at least partially by targeting miR-155-3p or miR-325-3p.^9,12,17^ We are also beginning to understand the mechanism through which macrophages in a proinflammatory/immunoregulatory state affect the differentiation of PDLSCs into cementoblasts,^9^ but the cellular and molecular events underlying regulation of inflammation-mediated cementogenesis are still unclear. New insights into the inflammation-mediated cementogenesis of cells are crucial for the design and development of next-generation regenerative therapeutics that can achieve integrated periodontal regeneration in an inflammatory environment, including the root cementum.

Mitochondria are a fundamental central modulator of successful cell differentiation and are one of the most vulnerable organelles during inflammation-induced cell dysfunction.^18–22^ However, the role of mitochondrial function and downstream signaling molecules in regulating inflammation-induced changes to the cementoblastic differentiation of PDLSCs remains unclear. In recent years, accumulating data have confirmed the importance of long noncoding RNA (lncRNA), an RNA molecule with a length between 200 and 100 000 nucleotides (nt) without protein-coding potential, in many physiological processes, such as organism development and cell differentiation.^23–27^ Importantly, lncRNAs have been found to actively participate in the regulation of mitochondrial bioenergetics or metabolism and are either located within or outside of the mitochondrial compartment.^28–30^ Unlike short microRNAs, lncRNAs are long enough to form a variety of intramolecular RNA-RNA interactions, which allows them to form a complex three-dimensional structure; the unique physical characteristics of lncRNAs allow their binding to mitochondrion-regulating proteins and the modulation of their stability, phosphorylation and/or relocalization.^28,31,32^ In this regard, we hypothesize that lncRNAs are potential active molecules that play a critical role in the damage to mitochondrial function and cementogenesis induced by inflammation.

To test our hypothesis and to investigate the underlying mechanism, we established an artificial inflammatory environment with tumor necrosis factor (TNF)-α plus interleukin-1 beta (IL-1β) to monitor the inflammation-induced changes in mitochondrial function and cementogenesis of PDLSCs. Mechanistically, we screened and identified the key lncRNAs involved in the cementoblastic differentiation and mitochondrial function of PDLSCs mediated by inflammation. Furthermore, potential proteins that can bind to the identified lncRNAs and modulate the mitochondrial bioenergetics and/or metabolism of PDLSCs were identified and verified. Our aim was to provide new insights into the cellular and molecular events underlying the cementogenesis of cells in an inflammatory environment and thus to provide new potential therapeutic targets for achieving cementum regeneration in future mainstream periodontal tissue engineering strategies.

## Results

### Inflammatory cytokines compromise the cementogenesis of PDLSCs

Human PDLSCs were successfully obtained from the permanent teeth of 7 donors (Fig. S1a), and their abilities to proliferate and differentiate into osteogenic, adipogenic and chondrogenic lineages were confirmed by colony-forming unit-fibroblast (CFU-F) assays (Fig. S1b), flow cytometric analysis (Fig. S1c), CCK-8 assays (Fig. S1d) and analyses of their multidifferentiation potential (Fig. S1e).

**Fig. 1:**
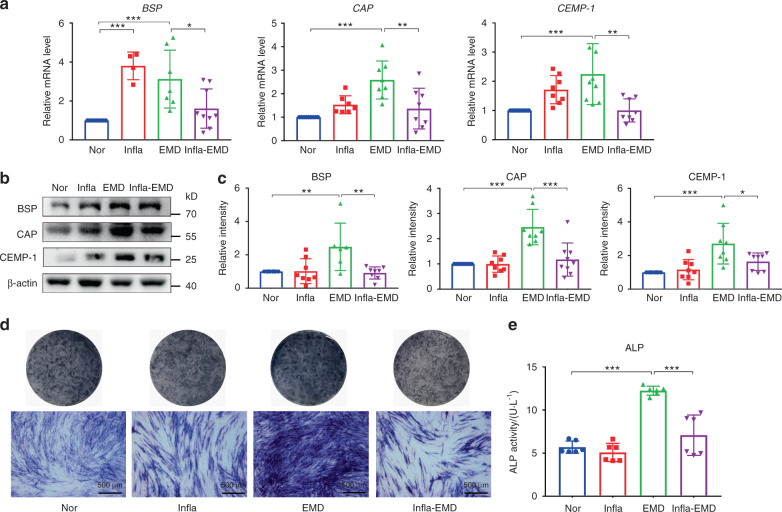
Inflammatory cytokines compromise the cementogenesis of PDLSCs. The cells were incubated in normal α-MEM (Nor), medium with the inflammatory cytokines TNF-α and IL-1β (Infla), medium with the cementoblastic inducer EMD (EMD), or medium with both inflammatory cytokines and EMD (Infla-EMD). **a** Relative cementoblastic differentiation-related gene expression levels of *BSP*, *CAP* and *CEMP-1* determined by qRT-PCR. **b** Relative cementoblastic differentiation-related protein expression of BSP, CAP and CEMP-1 determined by Western blots. **c** Semiquantitative analysis of protein expression levels (normalized to β-actin) in terms of relative gray density. **d** ALP staining (scale bar: 500 μm) of PDLSCs. **e** Quantification of ALP activity. The data are shown as the mean ± SD for *n* from 4 to 9; *P < 0.05, **P < 0.01 and ***P < 0.001 indicate significant differences between the indicated columns

Based on our previously established protocols,^9,16^ complete medium with 100 μg·mL^−1^ enamel matrix derivative (EMD, Emdogain®) was used to establish cementoblastic conditions for the induction of cementoblastic differentiation, and the addition of 10 ng·mL^−1^ TNF-α plus 5 ng·mL^−1^ IL-1β to the cell culture media created an inflammatory environment to identify how the presence of inflammatory cytokines influences the cementoblastic differentiation of cells. Here, PDLSCs incubated in normal and inflammatory environments were designated the Nor and Infla groups, respectively, and the EMD and Infla-EMD groups were created by addition of EMD to the aforementioned normal and inflammatory environments, respectively. After 7 days of induction, the cementoblastic differentiation potential of PDLSCs in each group was evaluated. Based on a quantitative real-time polymerase chain reaction (qRT-PCR) assay, the cells in the Infla-EMD group exhibited markedly lower expression levels of cell cementoblastic differentiation-related genes (*BSP*, *CAP* and *CEMP-1*) than the cells in the EMD group (Fig. 1a). Similarly, the incubation of cells in an inflammatory environment (Infla-EMD group) significantly decreased the levels of cell cementoblastic differentiation-related proteins (BSP, CAP and CEMP-1) (Western blot; Fig. 1b, c). Furthermore, the cells in the EMD group were more likely to show positive staining for alkaline phosphatase (ALP) (Fig. 1d) and exhibited higher cellular ALP activity than those in the Infla-EMD group (Fig. 1e).

To evaluate how inflammation influences cell cementogenesis in vivo, we incubated PDLSCs in a normal or inflammatory environment with EMD and 50 μg·mL^−1^ vitamin C (Vc) supplementation (EMD and Infla-EMD groups, respectively) for 14 days, and the obtained cell sheets combined with treated dentin matrix (TDM) were then subcutaneously transplanted into nude mice. At 8 weeks post-implantation, markedly higher CAP and CEMP-1 expression and more cementum-like tissue formation were observed in the transplants derived from the EMD group than in those from the Infla-EMD group (Fig. S2). Consistent with the in vitro data, the in vivo outcomes also indicate that inflammatory cytokines could compromise the cementogenesis of PDLSCs.

### Inflammatory cytokines cause mitochondrial dysfunction of PDLSCs during cementoblastic differentiation

Mitochondria, the major source of reactive oxygen species (ROS), reportedly play central roles in cell differentiation.^18,33^ Based on the reported literature,^34,35^ the levels of cellular ROS, mitochondrial ROS (mtROS), mitochondrial membrane potential (MMP), intracellular adenosine 5ʹ-triphosphate (ATP) content, mitochondrial DNA (mtDNA) content, mitochondrial morphology, mitochondrial respiratory chain complex and oxygen consumption rate (OCR) were used as vital indicators to assess the effects of inflammatory cytokines on the mitochondrial function of PDLSCs in the present study. Cellular ROS and mtROS levels were applied to identify an appropriate duration for detecting the mitochondrial function of PDLSCs, and the results revealed significantly different cellular ROS and mtROS levels between the Infla-EMD and EMD groups at Day 3 (Fig. S3 and S4). Therefore, a 3-day incubation period was selected to explore the mitochondrial function of PDLSCs in an inflammatory environment in the present study.

**Fig. 2:**
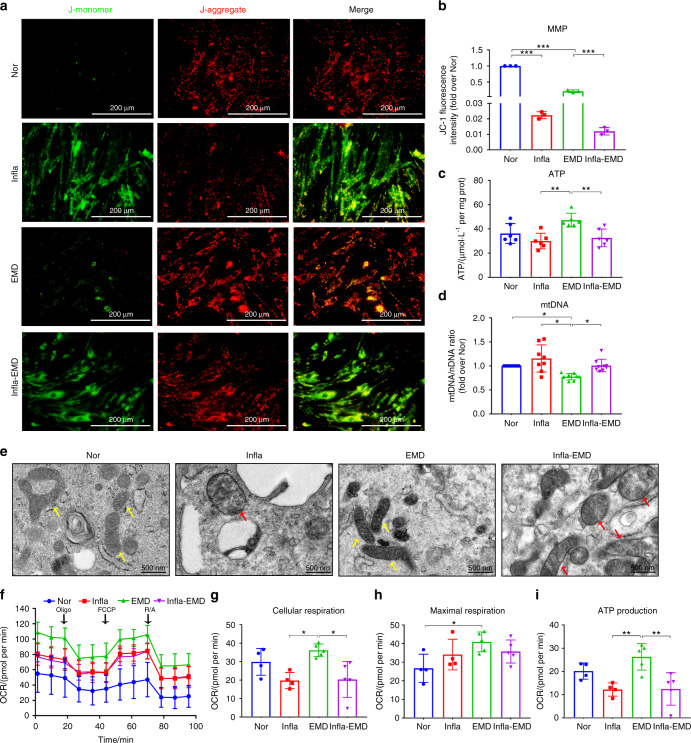
Inflammatory cytokines cause mitochondrial dysfunction in PDLSCs during cementoblastic differentiation. The cells were incubated in normal α-MEM (Nor), medium with the inflammatory cytokines TNF-α and IL-1β (Infla), medium with the cementoblastic inducer EMD (EMD), or medium with both inflammatory cytokines and EMD (Infla-EMD). **a** The MMP was determined using a JC-1 probe (immunofluorescence staining; scale bar: 200 μm). **b** Quantification of the MMP (reflected by the relative ratio of red/green fluorescence intensity of JC-1). **c** Intracellular ATP contents determined by ATP assays. **d** mtDNA content determined by qRT-PCR. **e** Morphometric analysis of mitochondria by transmission electron microscopy (yellow arrows indicate healthy mitochondria, red arrows indicate swollen mitochondria; scale bar: 500 nm). **f** The OCR of PDLSCs determined using a Seahorse Bioscience XF Analyzer; arrows indicate the sequential injection of the ATPase inhibitor Oligo (1 μmol·L^−1^), the uncoupling reagent carbonyl cyanide-p-trifluoromethoxyphenylhydrazone (FCCP, 1 μmol·L^−1^) and inhibitors of the electron transport chain rotenone/antimycin (R/A, 2 μmol·L^−1^). **g** Quantification of maximal respiration (differences between the maximum rate measurement after FCCP injection and the minimum rate measurement after R/A injection). **h** Quantification of cellular respiration (basic OCR value prior to Oligo injection). **i** Quantification of ATP production (difference between final rate measurement prior to Oligo injection and minimum rate measurement after Oligo injection). The data are shown as the mean ± SD for *n* from 3 to 8; *P < 0.05, **P < 0.01 and ***P < 0.001 indicate significant differences between the indicated columns

Consistent with the cellular ROS and mtROS levels, after a 3-day exposure to inflammatory cytokines, the PDLSCs from the Infla and Infla-EMD groups showed significant decreases in the MMP compared with those in the Nor and EMD groups, respectively (Fig. 2a and b). Similarly, the intracellular ATP content of the cells in the Infla-EMD group was significantly lower than that of the cells in the EMD group (Fig. 2c). However, markedly higher levels of mtDNA were found in the cells from the Infla-EMD group than in the cells from the EMD group (Fig. 2d). Because mitochondrial morphology is inherently correlated with its function,^36^ we observed mitochondria in the cells from the various groups by transmission electron microscopy. The mitochondria in the cells from the Infla and Infla-EMD groups exhibited significant morphological defects, including swelling and an altered mitochondrial matrix density (Fig. 2e). Because mitochondrial function or the capacity to supply energy through OXPHOS is key for cell differentiation,^37^ the OCR of the PDLSCs from the EMD and Infla-EMD groups was analyzed using a Seahorse Bioscience XF Analyzer. The data showed that incubation in an inflammatory environment (Infla-EMD group) markedly decreased the cellular respiration and mitochondrial ATP production of PDLSCs (Fig. 2f-i). However, in terms of maximal respiration, no significant difference was found between the PDLSCs from the EMD group and those from the Infla-EMD group (Fig. 2h). These data reveal that cells exhibit mitochondrial impairment and dysfunction in response to inflammatory incubation. Moreover, the expression of mitochondrial respiratory chain complex I/II in the PDLSCs in the Infla-EMD group was significantly lower than that in the EMD group (Fig. S5).

### Mitochondrial function is required for the cementoblastic differentiation of PDLSCs in either noninflammatory or inflammatory environments

**Fig. 3:**
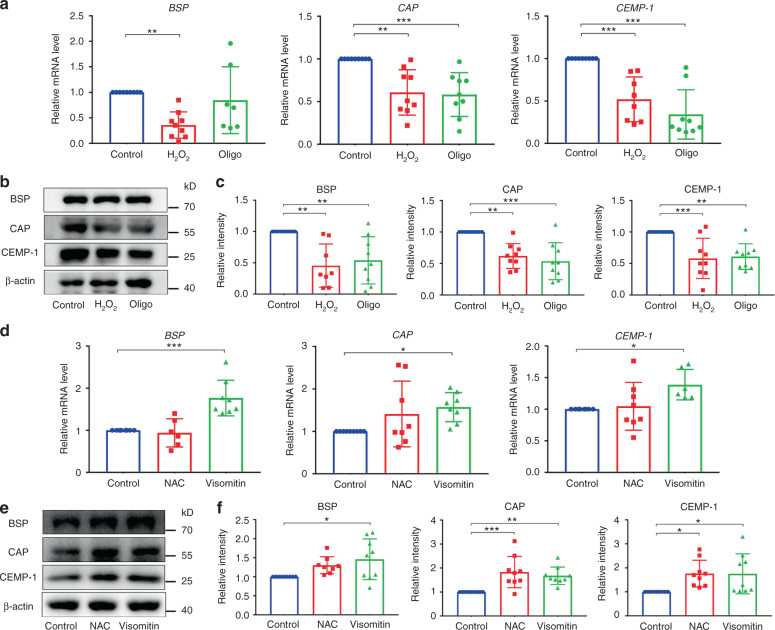
Mitochondrial function is required for the cementoblastic differentiation of PDLSCs in either noninflammatory or inflammatory environments. **a**–**c** Mitochondrial dysfunction impairs the cementoblastic differentiation of PDLSCs. The cells were incubated in medium with EMD (noninflammatory environment). **a** Relative cementoblastic differentiation-related gene expression levels of *BSP*, *CAP* and *CEMP-1* in the PDLSCs without (control) or with pretreatment with 10 μmol·L^−1^ H_2_O_2_ (H_2_O_2_) or 10 μg·mL^−1^ Oligo (Oligo) for 24 h (qRT-PCR). **b** Relative cementoblastic differentiation-related protein expression of BSP, CAP and CEMP-1 in the PDLSCs without (control) or with pretreatment with 10 μmol·L^−1^ H_2_O_2_ (H_2_O_2_) or 10 μg·mL^−1^ Oligo (Oligo) for 24 h (Western blot). **c** Semiquantitative analysis of the protein expression levels (normalized to β-actin) in terms of relative gray density. **d**–**f** Reversing inflammation-induced mitochondrial dysfunction rescued the cementoblastic differentiation of PDLSCs. The cells were incubated in medium with both inflammatory cytokines and EMD (inflammatory environment). **d** Relative cementoblastic differentiation-related gene expression levels of *BSP*, *CAP* and *CEMP-1* in the PDLSCs without (control) or with pretreatment with 1 mmol·L^−1^ NAC (NAC) or 20 nmol·L^−1^ Visomitin (Visomitin) for 2 h (qRT-PCR). **e** Relative cementoblastic differentiation-related protein expression of BSP, CAP and CEMP-1 in the PDLSCs without (control) or with pretreatment with 1 mmol·L^−1^ NAC (NAC) or 20 nmol·L^−1^ Visomitin (Visomitin) for 2 h (Western blot). **f** Semiquantitative analysis of protein expression levels (normalized to β-actin) in terms of relative gray density. The data are shown as the mean ± SD for *n* from 6 to 9; *P < 0.05, **P < 0.01 and ***P < 0.001 indicate significant differences between the indicated columns

To elucidate the role of mitochondrial function in the cementoblastic differentiation of PDLSCs, we observed how the changes in the mitochondrial function of cells correlate with cementoblastic differentiation in either a noninflammatory or an inflammatory environment. Based on the reported data^38–40^ and our own examinations (Fig. S6), PDLSCs were pretreated with an ROS generator (10 μmol·L^−1^ H_2_O_2_) or mitochondrial inhibitor (10 μg·mL^−1^ oligomycin, Oligo) for 24 h to inhibit cell mitochondrial function, whereas the cells were incubated for 2 h with an ROS scavenger (1 mmol·L^−1^ N-acetylcysteine, NAC) or mitochondrial antioxidant (20 nmol·L^−1^ Visomitin) to enhance the mitochondrial function of the cells. As demonstrated by qRT-PCR and Western blot assays, treatment with either H_2_O_2_ or Oligo significantly decreased the expression of cementoblastic differentiation-related genes/proteins in the PDLSCs incubated in medium with EMD (a noninflammatory environment) (Fig. 3a–c). In contrast, we found that reversing inflammation-induced mitochondrial dysfunction with NAC or Visomitin rescued the cementoblastic differentiation of PDLSCs (Fig. 3d–f). Thus, mitochondrial function is closely correlated with the cementoblastic differentiation of PDLSCs, regardless of whether the cells were incubated in a noninflammatory or an inflammatory environment. The relationship between mitochondrial function and cell cementogenesis was further confirmed by clinical evidence, which showed that periodontitis-induced cementum destruction was accompanied by higher mtROS and intracellular ROS levels in the gingival tissues derived from periodontally diseased teeth than in the gingival tissues of healthy teeth (Fig. S7).

### Identification and validation of *gastric cancer associated transcript 2* (*GACAT2*) as a key lncRNA associated with the cementoblastic differentiation and mitochondrial function of PDLSCs

**Fig. 4:**
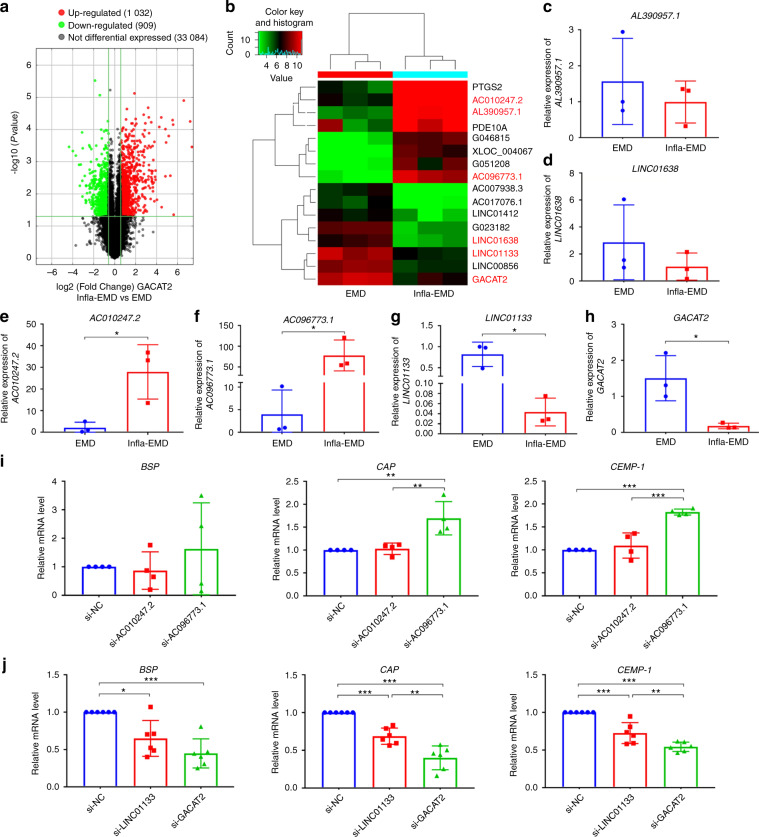
Identification and validation of *GACAT2* as a key lncRNA associated with the cementoblastic differentiation of PDLSCs. **a–h** Identification of differentially expressed lncRNAs between the PDLSCs incubated in noninflammatory environments and the PDLSCs incubated in inflammatory environments. The cells were incubated in medium with the cementoblastic inducer EMD alone (EMD) or EMD plus inflammatory cytokines (Infla-EMD). **a** Volcano plots of the differentially expressed (fold change >1.5 and adjusted *P* < 0.05) lncRNAs in the PDLSCs between the EMD and Infla-EMD groups. **b** Heatmap of the top 8 lncRNAs with upregulated expression and the top 8 lncRNAs with downregulated expression in the PDLSCs of the EMD group compared with those in the Infla-EMD group. Six lncRNAs, *AL390957.1* (**c**), *LINC01638* (**d**), *AC010247.2* (**e**), *AC096773.1* (**f**), *LINC01133* (**g**), and *GACAT2* (**h**), were selected for qRT-PCR verification according to their RNA length (<2 000 nt), intensity (>100), relation (without overlapping with coding transcripts) and database source (in the GENCODE or RefSeq public databases), and 10 other lncRNAs were excluded. **c** and **d** No significant changes in *AL390957.1* and *LINC01638* expression were observed in the cells incubated in the noninflammatory or inflammatory environments. **e** and **f** *AC010247.2* and *AC096773.1* expression was significantly increased in an inflammatory environment. **g** and **h** *LINC01133* and *GACAT2* expression was significantly decreased in an inflammatory environment. **i** The inhibition of *AC096773.1* (si-*AC096773.1*), but not *AC010247.2* (si-*AC010247.2*), increased the relative cementoblastic differentiation-related gene expression levels of *BSP*, *CAP* and *CEMP-1* in the PDLSCs incubated in an inflammatory environment (qRT-PCR). **j** The inhibition of *LINC01133* (si-*LINC01133*) or *GACAT2* (si-*GACAT2*) decreased the relative cementoblastic differentiation-related gene expression levels of *BSP*, *CAP* and *CEMP-1* in the PDLSCs incubated in a noninflammatory environment (qRT-PCR); the most significant change was observed in response to *GACAT2* inhibition. The data are shown as the mean ± SD for *n* from 3 to 6; *P < 0.05, **P < 0.01 and ***P < 0.001 indicate significant differences between the indicated columns

Given that lncRNAs are key regulators in cell differentiation,^24,25,27,41^ differentially expressed lncRNAs (*P* < 0.05 and fold change > 1.5) between the PDLSCs incubated in a noninflammatory environment (EMD group) and those incubated in an inflammatory environment (Infla-EMD group) were identified by lncRNA microarray analysis. As a result, 1 032 lncRNAs were shown to have significantly upregulated expression and 909 lncRNAs had significantly downregulated expression in the cells from the Infla-EMD group compared with those from the EMD group (Fig. 4a), and the names of the top 8 lncRNAs with upregulated expression and the top 8 with downregulated expression were presented in the heatmap in Fig. 4b. From the 16 lncRNAs, *AL390957.1*, *LINC01638*, *AC010247.2*, *AC096773.1*, *LINC01133* and *GACAT2* had an appropriate RNA length (< 2,000 nt), had a raw signal intensity >100, did not overlap with coding transcripts and were included in a database (in GENCODE or RefSeq public databases); thus, these lncRNAs were selected (Table S1) and then subjected to qRT-PCR verification, and 10 other lncRNAs were excluded. Based on the qRT-PCR assay, only 4 lncRNAs (*AC010247.2*, *AC096773.1*, *LINC01133* and *GACAT2*) were in fact differentially expressed between the EMD and Infla-EMD groups (Fig. 4e-h), whereas no significant changes in the *AL390957.1* and *LINC01638* expression levels were found between the two groups (Fig. 4c, d). Thus, *AC010247.2*, *AC096773.1*, *LINC01133* and *GACAT2* were selected for further investigation, and the other 2 lncRNAs (*AL390957.1* and *LINC01638*) were excluded.

To identify which of the 4 selected lncRNAs were linked to cementoblastic differentiation, we determined the changes in cementoblastic differentiation-related gene expression in PDLSCs in response to the knockdown of each lncRNA. Given that the expression of *AC010247.2* and *AC096773.1* was significantly increased in an inflammatory environment (Fig. 4e, f), small interfering RNAs (siRNAs) were used to silence their expression in cells incubated in an inflammatory environment. In contrast, *LINC01133* and *GACAT2* expression was significantly decreased in an inflammatory environment (Fig. 4g, h), and siRNAs were used to silence the expression of *LINC01133* and *GACAT2* in a noninflammatory environment. After the knockdown efficacy of each lncRNA (*AC010247.2*, *AC096773.1*, *LINC01133* or *GACAT2*) in PDLSCs was validated (Fig. S8), the expression of cementoblastic differentiation-related genes in the PDLSCs transfected with the corresponding siRNAs was evaluated by qRT-PCR. For the 4 potential target lncRNAs, the inhibition of *LINC01133* or *GACAT2* significantly influenced the expression levels of all cementoblastic differentiation-related genes (*BSP*, *CAP* and *CEMP-1*), whereas the inhibition of *AC096773.1* only affected *CAP* and *CEMP-1* expression in PDLSCs, and the inhibition of *AC010247.2* did not affect the expression of any cementoblastic differentiation-related genes (Fig. 4i, j). Hence, in this study, we concluded that *LINC01133* and *GACAT2* were closely related to cell cementoblastic differentiation. The comparison of gene expression in the PDLSCs transfected with si-*GACAT2* or si-*LINC01133* showed that si-*GACAT2* transfection led to lower levels of cementoblastic differentiation-related genes (vs. si-*LINC01133* transfection) (Fig. 4j). Therefore, *GACAT2* was ultimately selected as the key cementoblastic differentiation-related lncRNA for further gain/loss-of-function studies of both the mitochondrial function and cementoblastic differentiation of PDLSCs and in-depth mechanistic investigation. Here, the clinical significance of *GACAT2* in periodontitis was indirectly demonstrated by the lower expression of *GACAT2* in gingival tissues from teeth with periodontitis than in those from healthy tissues (Fig. S9). Moreover, no significant changes in the *GACAT2* expression levels were found between the PDLSCs from the Nor group and those from the EMD group (Fig. S10), indicating that *GACAT2* was mainly influenced by an inflammatory environment during cementoblastic differentiation.

To identify whether *GACAT2* is also correlated with mitochondrial function, we investigated how *GACAT2* knockdown affected the mitochondrial function of PDLSCs. The mtROS levels of the cells transfected with si-*GACAT2* were markedly higher than those of the cells transfected with si-NC (Fig. S11a, b). Consistently, *GACAT2* knockdown decreased ATP, MMP, and mtDNA levels and the expression of mitochondrial respiratory chain complex II/III/IV/V in PDLSCs (Fig. S11c–h). Additionally, the inhibition of *GACAT2* reduced the cellular respiration and mitochondrial ATP production of PDLSCs, which suggested that the ablation of *GACAT2* elicits mitochondrial dysfunction in PDLSCs (Fig. S11i-l).

### *GACAT2* overexpression reverses inflammation-compromised cementogenesis and mitochondrial function of PDLSCs

**Fig. 5:**
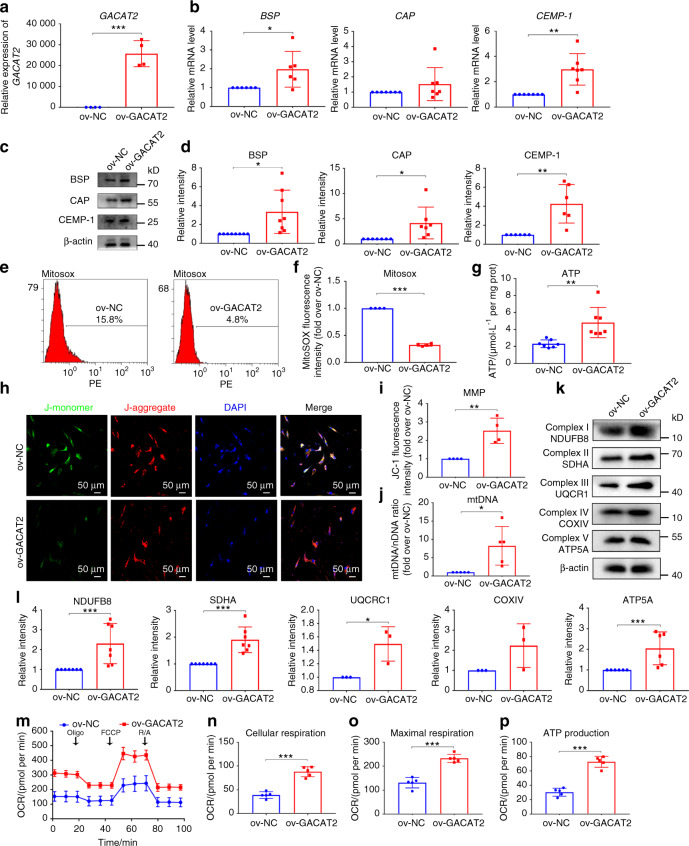
Overexpression of *GACAT2* (ov-*GACAT2*) reverses the inflammation-compromised cementoblastic differentiation and mitochondrial function of PDLSCs. The cells were incubated in medium with both inflammatory cytokines and EMD (inflammatory environment). **a** Overexpression efficiency of *GACAT2* in PDLSCs validated by qRT-PCR. **b** Relative cementoblastic differentiation-related gene expression levels of *BSP*, *CAP* and *CEMP-1* determined by qRT-PCR. **c** Relative cementoblastic differentiation-related protein expression of BSP, CAP and CEMP-1 determined by Western blots. **d** Semiquantitative analysis of protein expression levels (normalized to β-actin) in terms of relative gray density. **e** mtROS levels in PDLSCs determined with the aid of a MitoSOX probe (flow cytometric analysis). **f** Quantification of mtROS levels (reflected by the relative fluorescence intensity of MitoSOX). **g** Intracellular ATP contents (ATP assay). **h** The MMP was determined with a JC-1 probe (immunofluorescence staining; scale bar: 50 μm). **i** Quantification of MMP levels (reflected by the relative ratio of red/green fluorescence intensity of JC-1). **j** mtDNA content determined by qRT-PCR. **k** Relative mitochondrial complex-related protein expression of NDUFB8 (subunit of complex I), SDHA (subunit of complex II), UQCRC1 (subunit of complex III), COXIV (subunit of complex IV) and ATP5A (subunit of complex V) determined by Western blots. **l** Semiquantitative analysis of protein expression levels (normalized to β-actin) in terms of relative gray density. **m** OCR of PDLSCs determined by a Seahorse Bioscience XF Analyzer; arrows indicate the sequential injection of 1 μmol·L^−1^ Oligo, 1 μmol·L^−1^ FCCP and 2 μmol·L^−1^ R/A. **n** Quantification of cellular respiration (basic OCR value prior to Oligo injection). **o** Quantification of maximal respiration (differences between the maximum rate measurement after FCCP injection and the minimum rate measurement after R/A injection). **p** Quantification of ATP production (difference between the final rate measurement prior to Oligo injection and the minimum rate measurement after Oligo injection). The data are shown as the mean ± SD for *n* from 3 to 8; *P < 0.05, **P < 0.01 and ***P < 0.001 indicate significant differences between the indicated columns

Given that *GACAT2* knockdown negatively affected the mitochondrial function and cementoblastic differentiation of PDLSCs in a noninflammatory environment, we then investigated how *GACAT2* overexpression affected cells incubated in an inflammatory environment. Here, we overexpressed *GACAT2* in the PDLSCs with lentivirus carrying *GACAT2* cDNA (ov-*GACAT2*) (Fig. 5a) and then incubated the above stable overexpression cells in medium with both inflammatory cytokines and EMD (inflammatory environment). Compared with ov-NC transfection, ov-*GACAT2* transfection led to significantly higher *BSP* and *CEMP-1* gene expression levels (qRT-PCR; Fig. 5b) and significantly higher BSP, CAP and CEMP-1 protein expression levels in PDLSCs (Western blot; Fig. 5c, d). In addition, *GACAT2* overexpression decreased cell mtROS production (Fig. 5e, f) and induced increases in cell ATP production, MMP, mtDNA content and the expression of mitochondrial respiratory chain complex I/II/III/V (Fig. 5g-l). Similarly, the cellular respiration, maximal respiration and mitochondrial ATP production of PDLSCs determined by OCR assays increased significantly following *GACAT2* overexpression (Fig. 5m-p). The cells incubated in an inflammatory environment and transfected with ov-*GACAT2* or ov-NC were subcutaneously transplanted into male nude mice, and the cells with stable *GACAT2* expression incubated in an inflammatory environment showed increased cementogenesis-related marker expression in transplants (Fig. S12a-d) and indeed resulted in more cementum-like tissue formation on the surface of TDM bioscaffolds (Fig. S12e). Together, these data suggest that *GACAT2* overexpression can reverse the inflammation-compromised cementogenesis and mitochondrial function of PDLSCs.

### Identification and validation of pyruvate kinase M1/2 (PKM1/2) as a direct binding protein of *GACAT2* that influences mitochondrial function

**Fig. 6:**
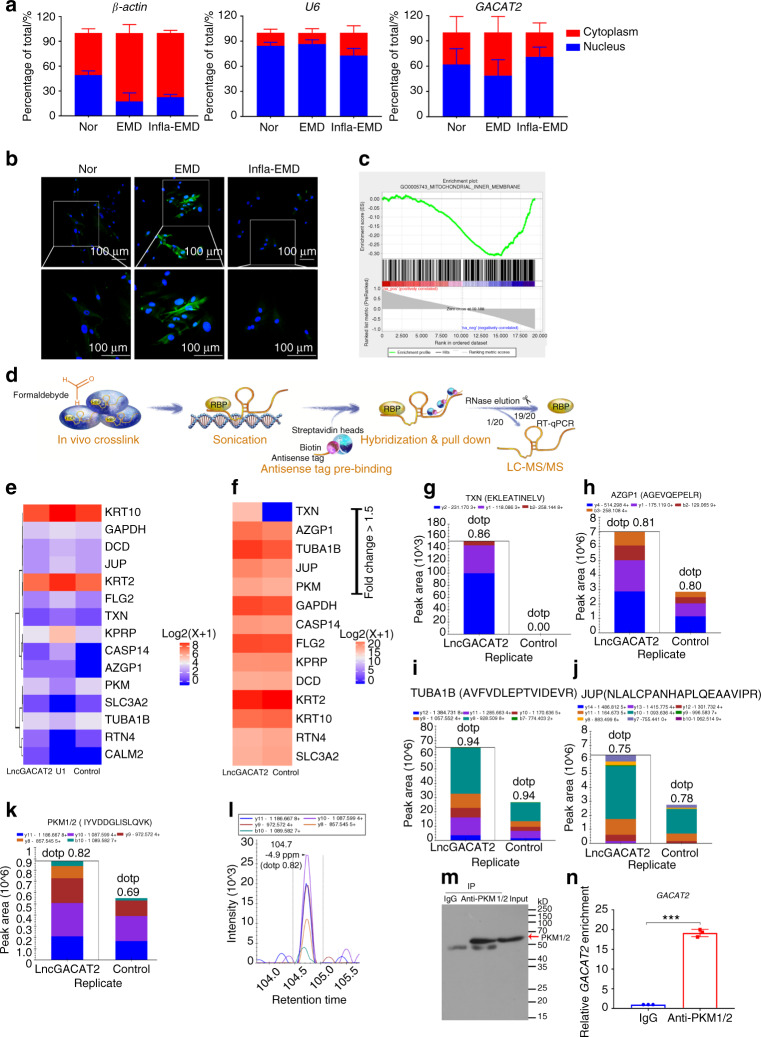
Identification and validation of PKM1/2 as a direct binding protein of *GACAT2* that influences mitochondrial function. **a** and **b** Determination of *GACAT2* localization in both the cytoplasm and nucleus of PDLSCs. The cells were incubated in normal α-MEM (Nor), medium with the cementoblastic inducer EMD (EMD), or medium with both inflammatory cytokines and EMD (Infla-EMD). **a** Percentages of nuclear and cytoplasmic *GACAT2* determined by qRT-PCR (*U6* and *β-actin* served as the nuclear and cytoplasmic controls, respectively). **b** Subcellular localization of *GACAT2* determined by the RNA SCOPE assay (scale bar: 100 μm). **c** *GACAT2* expression was negatively correlated with mitochondrial inner membrane-associated gene signatures (GSEA). **d** The ChIRP method was applied to screen the potential proteins binding to *GACAT2*. **e** Heatmap of 15 *GACAT2*-binding proteins screened by ChIRP-MS assays (unique peptide ≥2, fold change >1.2); U1 probes were used as the positive control (U1), and nontargeting probes (control) were used as the negative control. **f** Quantification of labeled reference peptides for *GACAT2*-binding proteins (PRM assay, nontargeting probes were used as the negative control). **g**–**k** Product ion pattern of the selected proteins (fold change >1.5), i.e., TXN (**g**), AZGP1 (**h**), TUBA1B (**i**), JUP (**j**) and PKM1/2 (**k**) (PRM assay; EKLEATINELV for the TXN protein, AGEVQEPELR for the AZGP1 protein, AVFVDLEPTVIDEVR for the TUBA1B protein, NLALCPANHAPLQEAAVIPR for the JUP protein, and IYVDDGLISLQVK for the PKM1/2 protein); nontargeting probes were used as controls; different colors indicate different fragment ions from the same polypeptide, and each peptide was quantified using fragment ions. **l** Chromatograph of a labeled peptide (IYVDDGLISLQVK) for the PKM1/2 protein; PKM1/2 was selected for further verification because it is more closely related to mitochondrial function than AZGP1, TUBA1B and JUP (based on information arising from the UniProt Database), and TXN was excluded from further investigation due to its inappropriate chromatography (Fig. S15). **m** and **n** Confirmation of the interaction between PKM1/2 and *GACAT2* by RIP assays. The PKM1/2 protein immunoprecipitated by the anti-PKM1/2 antibody was verified by immunoblotting analysis (**m**), and the enrichment of *GACAT2* immunoprecipitated by anti-PKM1/2 antibodies was determined by RIP assays (**n**) (IgG antibodies were used as the control). The data are shown as the mean ± SD for *n* = 3; ***P < 0.001 indicate significant differences between the indicated columns

Given that the functions of lncRNAs are closely linked to their subcellular localization,^42,43^ the subcellular localization of *GACAT2* in PDLSCs was determined. RNA extracted from nuclear and cytoplasmic fractions was successfully identified using a nuclear marker (*U6*) and cytoplasmic marker (*β-actin*), and the nuclear and cytoplasmic expression of *GACAT2* in the PDLSCs from the Nor, EMD and Infla-EMD groups was then detected by qRT-PCR. The results revealed that *GACAT2* was localized in both the cytoplasm and nucleus of PDLSCs (Fig. 6a). Similar outcomes were observed with cells detected using RNAscope® in situ RNA detection technology (RNAscope® ISH), which showed that *GACAT2* was localized in both the cytoplasm and the nucleus of PDLSCs (Fig. 6b). Furthermore, mitochondrial fractionation experiments demonstrated that *GACAT2* was located in the mitochondria of PDLSCs (Fig. S13).

According to the literature,^26,41,44–46^ lncRNAs in the cytoplasm and nucleus could fulfill their functions by interacting with microRNAs (miRNAs) or proteins; thus, we subsequently screened *GACAT2*-binding mitochondrial function-related molecules. First, gene set enrichment analysis (GSEA) revealed that *GACAT2* expression was negatively related to the mitochondrial inner membrane-associated gene signatures of PDLSCs (Fig. 6c; *P* < 0.05, FDR < 0.25), which was consistent with our previous findings that *GACAT2* plays an essential role in regulating mitochondrial function. Subsequently, we screened *GACAT2*-binding miRNAs and proteins using competing endogenous RNA (ceRNA) analysis and comprehensive identification of RNA-binding proteins by mass spectrometry (ChIRP-MS), respectively. The ceRNA network constructed by miRNA target prediction software (based on TargetScan and miRanda) showed that 10 mRNAs may be the final target of *GACAT2* when lncRNA functions as a “sponge” to sequester miRNAs (Table S2), and qRT-PCR demonstrated that only 3 mRNAs*—*type-1 angiotensin II receptor-associated protein (AGTRAP), zinc finger protein 672 (ZNF672) and calmodulin-like protein 3 (CALML3)—were differentially expressed in the PDLSCs transfected with ov-NC or ov-*GACAT2* (Fig. S14). However, none of these molecules was reported to be tightly related to mitochondrial function. Thus, we did not further investigate *GACAT2*-binding miRNAs and speculated that *GACAT2* may function by interacting with proteins.

Next, a ChIRP-MS assay was performed to screen for potential *GACAT2*-binding proteins (Fig. 6d). After an initial screening based on unique peptides (≥2) and fold change (> 1.2), ChIRP-MS analysis revealed that 15 proteins were enriched in the ChIRP lysate (Fig. 6e and Table S3). Furthermore, parallel reaction monitoring (PRM) was used to verify and quantify the labeled reference peptides of the screened *GACAT2*-binding proteins (Fig. 6f and Table S4). According to their fold change (>1.5), 5 proteins—thioredoxin (TXN), zinc-binding protein (AZGP1), tubulin alpha 1b (TUBA1B), junction plakoglobin (JUP), and PKM1/2—were selected as candidates for subsequent validation (Fig. 6g-l); TXN was excluded due to its inappropriate chromatography (Fig. S15). Among the 4 other candidates, PKM1/2 was ultimately selected for detailed testing because it is reportedly more closely related to mitochondrial function than AZGP1, TUBA1B and JUP.^47,48^ In addition, the close link between PKM1/2 and mitochondrial function is documented in the UniProt Database. The interaction of *GACAT2* with PKM1/2 was verified by RNA immunoprecipitation (RIP) using anti-PKM1/2 and isotype immunoglobulin G (IgG) antibodies (control). As expected, PKM1/2 proteins were successfully precipitated by the anti-PKM1/2 antibody (Fig. 6m), and *GACAT2* was significantly enriched in the PKM1/2-precipitated group compared with the IgG (control) group (Fig. 6n). Taken together, our results demonstrate that PKM1/2 is a direct binding protein of *GACAT2* that influences the mitochondrial function of PDLSCs.

### Overexpression/Inhibition of *GACAT2* affects the expression, activity, allosteric regulation, post-translational modification and translocation of PKM1/2

**Fig. 7:**
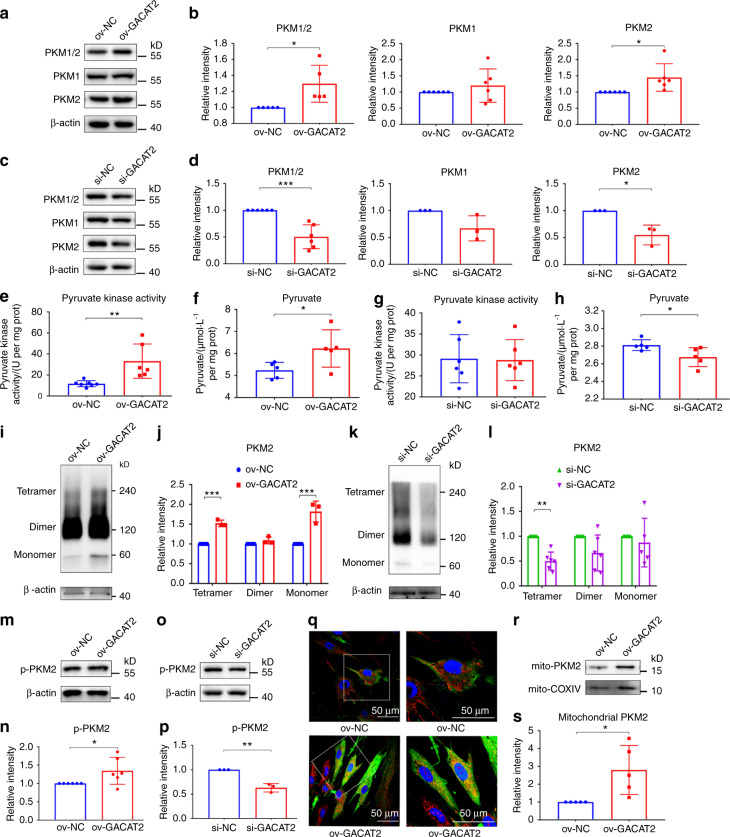
Overexpression/inhibition of *GACAT2* affects the expression, activity, allosteric regulation, post-translational modification and translocation of PKM1/2. **a**–**d** Relative protein expression levels of PKM1/2, PKM1 and PKM2 in PDLSCs (with overexpression or inhibition of *GACAT2*) determined by Western blots and semiquantitative analysis of their expression levels (normalized to β-actin) in terms of relative gray density. The cells were transfected with ov-NC or ov-*GACAT2* and incubated in medium with both inflammatory cytokines and EMD (inflammatory environment) (**a** and **b**) or were transfected with si-NC or si-*GACAT2* and incubated in medium with the cementoblastic inducer EMD (noninflammatory environment) (**c** and **d**). **e**–**h** PK activity and production of pyruvate in PDLSCs (with the overexpression or inhibition of *GACAT2*). The cells were transfected with ov-NC or ov-*GACAT2* and incubated in an inflammatory environment (**e** and **f**) or were transfected with si-NC or si-*GACAT2* and incubated in a noninflammatory environment (**g** and **h**). **i**–**l** Tetramer, dimer and monomer levels of PKM2 in PDLSCs (with overexpression or inhibition of *GACAT2*) determined by western blots (**i** and **k**) and semiquantitative analysis of their expression levels (normalized to β-actin) in terms of relative gray density (**j** and **l**). The cells were transfected with ov-NC or ov-*GACAT2* and incubated in an inflammatory environment (**i** and **j**) or were transfected with si-NC or si-*GACAT2* and incubated in a noninflammatory environment (**k** and **l**). **m**–**p** p-PKM2 in PDLSCs (with overexpression or inhibition of *GACAT2*) was determined by western blots and semiquantitative analysis of their expression levels (normalized to β-actin) in terms of relative gray density. The cells were transfected with ov-NC or ov-*GACAT2* and incubated in an inflammatory environment (**m** and **n**) or were transfected with si-NC or si-*GACAT2* and incubated in a noninflammatory environment (**o** and **p**). **q** Immunofluorescence staining for mitochondria (MitoTracker, red), PKM2 (green) and DAPI (blue) in the PDLSCs transfected with ov-NC or ov-*GACAT2* and incubated in an inflammatory environment (scale bar: 50 μm). **r** and **s** Relative protein expression levels of mitochondrial PKM2 in PDLSCs (with overexpression of *GACAT2*) determined by Western blots (**r**) and semiquantitative analysis of their expression levels (normalized to COXIV) in terms of relative gray density (**s**). The data are shown as the mean ± SD for *n* from 3 to 6; *P < 0.05, **P < 0.01 and ***P < 0.001 indicate significant differences between the indicated columns

We investigated how *GACAT2* affects mitochondrial function by binding to PKM1/2 and found that the overexpression of *GACAT2* could increase the protein expression levels of PKM1/2 and PKM2 (Fig. 7a, b), whereas *GACAT2* knockdown decreased PKM1/2 and PKM2 expression (Fig. 7c, d), even though *GACAT2* and *PKM1/2* did not appear to affect each other at the gene level (Fig. S16). Given that pyruvate kinase (PK) can catalyze the last step of glycolysis and convert phosphoenolpyruvate to pyruvate,^47,49^ PK activity and the production of pyruvate were detected as indicators of mitochondrial metabolism. *GACAT2* overexpression elevated PK activity and pyruvate production, whereas *GACAT2* knockdown decreased pyruvate production (Fig. 7e-h). Furthermore, a disuccinimidyl suberate (DSS) crosslinking study coupled with a Western blot assay showed that *GACAT2* overexpression increased the formation of PKM2 tetramers, which are the most enzymatically active form, to exert biological functions (Fig. 7i-l). PKM2 phosphorylation on tyrosine 105 (p-PKM2), an indicator of monomer/dimer formation,^50^ was then measured by Western blots. The overexpression and silencing of *GACAT2* increased and decreased the protein expression of p-PKM2, respectively (Fig. 7m-p). Because PKM2 mitochondrial translocation also contributes to the regulation of mitochondrial function,^47,51,52^ our further experiments based on immunofluorescence staining with an anti-PKM1/2 antibody and MitoTracker (mitochondrial marker) showed that *GACAT2* overexpression promoted the mitochondrial translocation of PKM2 (Fig. 7q), which was consistent with the results of a mitochondrial fractionation analysis, showing that PKM2 was increased in the mitochondrial fraction of PDLSCs after *GACAT2* overexpression (Fig. 7r, s).

### Inhibition of PKM1/2 impairs *GACAT2* overexpression-rescued mitochondrial function and cementoblastic differentiation of PDLSCs

**Fig. 8:**
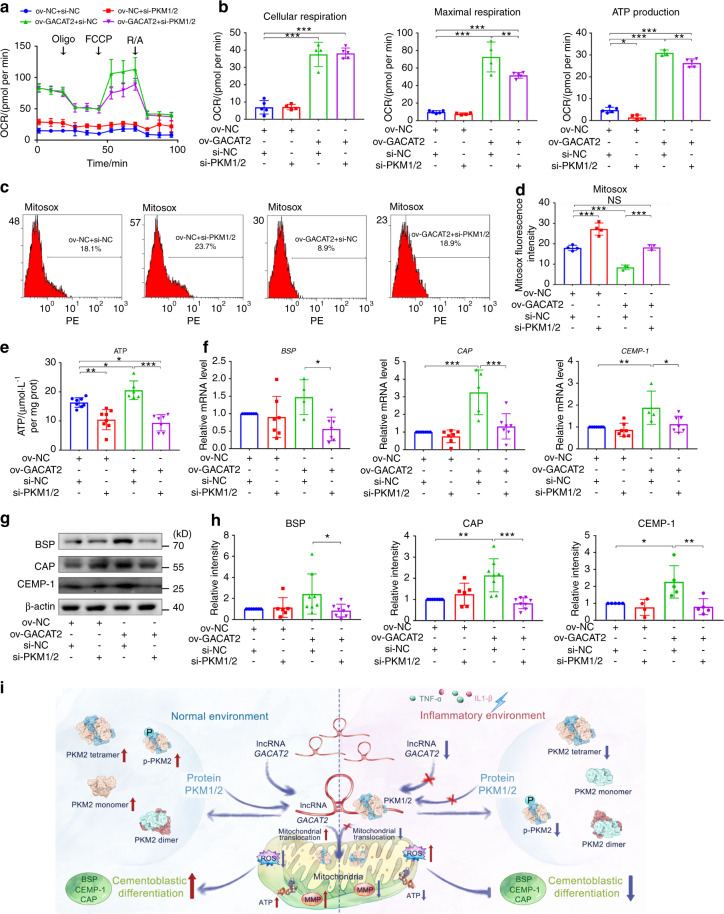
Inhibition of PKM1/2 impairs *GACAT2* overexpression-rescued mitochondrial function and cementoblastic differentiation of PDLSCs. The cells were transfected with ov-NC or ov-*GACAT2* and incubated in medium with both inflammatory cytokines and EMD (inflammatory environment), and both ov-NC- and *GACAT2*-transfected cells were then further transfected with si-NC or si-PKM1/2. **a** OCR of PDLSCs determined by a Seahorse Bioscience XF Analyzer; arrows indicate the sequential injection of 1 μmol·L^−1^ Oligo, 1 μmol·L^−1^ FCCP and 2 μmol·L^−1^ R/A. **b** Quantification of cellular respiration (basic OCR value prior to Oligo injection), maximal respiration (differences between the maximum rate measurement after FCCP injection and the minimum rate measurement after R/A injection) and ATP production (difference between the final rate measurement prior to Oligo injection and the minimum rate measurement after Oligo injection) of PDLSCs. **c** mtROS levels in PDLSCs determined with the aid of a MitoSOX probe (flow cytometric analysis). **d** Quantification of mtROS levels (reflected by the relative fluorescence intensity of MitoSOX). **e** Intracellular ATP contents (ATP assay). **f** Relative cementoblastic differentiation-related gene expression levels of *BSP*, *CAP* and *CEMP-1* determined by qRT-PCR. **g** Relative cementoblastic differentiation-related protein expression of BSP, CAP and CEMP-1 determined by western blots. **h** Semiquantitative analysis of protein expression levels (normalized to β-actin) in terms of the relative gray density. **i** The diagram shows that the lncRNA *GACAT2* plays a central role in regulating mitochondrial function and cementoblastic differentiation of PDLSCs in an inflammatory environment. Mechanistically, it functions by binding to PKM1/2 proteins to modulate cell mitochondrial bioenergetics and/or metabolism. The data are shown as the mean ± SD for *n* from 3 to 8; *P < 0.05, **P < 0.01 and ***P < 0.001 indicate significant differences between the indicated columns

To determine whether binding to PKM1/2 is needed for *GACAT2*-mediated regulation of cell mitochondrial function and cementoblastic differentiation, we silenced PKM1/2 with siRNAs (Fig. S16c) under *GACAT2*-overexpressing conditions. Functional studies revealed that *GACAT2* overexpression ameliorated mitochondrial dysfunction (i.e., OCR, mtROS and ATP levels) in the Infla-EMD group; this function was abolished by transfection with si-PKM1/2 in PDLSCs (Fig. 8a–e). Similarly, the analysis of cementoblastic differentiation markers (BSP, CAP and CEMP-1) by qRT-PCR and Western blots (Fig. 8f–h) revealed that the inhibition of PKM1/2 could abolish the *GACAT2* overexpression-induced increase in the cementoblastic differentiation of PDLSCs. Thus, the function of *GACAT2* in reversing the damage to mitochondrial function and cementoblastic differentiation induced by inflammation is primarily dependent on its binding to the PKM1/2 protein.

## Discussion

Periodontitis, a leading cause of tooth loss in adults, causes progressive destruction of the tooth-supporting apparatus, which is composed of at least three vital tissues that function together as a unit to fix the tooth to the jawbone and then to exert the appropriate chewing function.^1,2,6^ Because the cementum attaches PDL collagen fibers to the root surface, cementogenesis is an important prerequisite for any therapeutic paradigm that aims to restore the lost/damaged tooth-supporting structures.^6,53^ Although PDLSCs can give rise to the cementoblastic lineage, they inevitably show a compromised function within a diseased periodontium due to the inflammatory environment and subsequently cannot properly differentiate into cementoblasts, the only cell type that can grow cementum tissue.^9,12^ Here, we aimed to identify whether inflammation-compromised cementoblastic differentiation is linked to cell mitochondrial dysfunction and to monitor the key lncRNAs involved in cellular events during cementoblastic differentiation. We also investigated how these molecules modulate mitochondrial bioenergetics and/or metabolism and identified new therapeutic targets for combating inflammation-induced cell dysfunction.

Since BSP, CAP, CEMP-1, and ALP activity are known to play essential roles in regulating mineral formation, including cementum,^9,54–57^ they were used as cementogenesis-related markers in the present study. Similar to our reported protocols,^9^ EMD was used as an inducer for cell cementoblastic differentiation, and the use of EMD for cementoblastic induction is supported by its well-documented ability to promote cementoblastic differentiation in cell research and to guide new cementum formation in experimental animals.^58^ TNF-α and IL-1β are essential in periodontal destruction, represent proinflammatory cytokines that participate in the initiation and progression of periodontitis^59,60^ and hence have been used to simulate an inflammatory environment in our previous studies^13,14,16^ as well as in the present study. Consistent with previous findings that inflammatory cytokines, including TNF-α and IL-1β, may inhibit the cementoblastic and osteogenic differentiation of cells,^9,12,14,15^ an adverse effect of inflammation on the cementoblastic differentiation of PDLSCs was demonstrated in this study at the protein and gene levels (Fig. 1). Because mitochondrial dysfunction/oxidative stress is a pathogenic mechanism of many, if not all, chronic inflammatory diseases, such as periodontitis, and because mitochondria are indeed involved in many physiological processes, such as cell differentiation,^18,19,22^ we hypothesized that mitochondrial function could contribute to PDLSC cementoblastic differentiation in either normal or inflammatory environments.

To test this hypothesis, we detected a series of indicators, *e.g*., ROS levels, MMP, ATP production, mtDNA content, mitochondrial morphology, mitochondrial respiratory chain complex and OCR, to investigate the effects of inflammatory cytokines on PDLSC mitochondrial function. Unsurprisingly, we found that inflammatory cytokines elicited mitochondrial dysfunction in PDLSCs (Fig. 2, S3, S4 and S5), which is consistent with previous findings that TNF-α plus IFN-γ or IL-1β enhances ROS concentrations, inhibits mitochondrial respiratory chain complex I and/or damages mitochondrial activity, and these effects cause cell apoptosis or cartilage degradation.^61,62^ Furthermore, the effect of mitochondrial function on the cementoblastic differentiation of PDLSCs in an inflammatory environment was identified using an ROS generator/mitochondrial inhibitor or ROS scavenger/mitochondrial antioxidant. The results showed a link between mitochondrial dysfunction and compromised cementoblastic differentiation of PDLSCs, and more interestingly, we demonstrated that reversing inflammation-induced mitochondrial dysfunction could rescue the cementoblastic differentiation of PDLSCs (Fig. 3). These data indicate that the mitochondrial dysfunction of PDLSCs in an inflammatory environment is a main reason for their compromised cementoblastic differentiation and thus identify a new paradigm for understanding cell cementogenesis, but the downstream signaling molecules that regulate the inflammation-induced changes to the mitochondrial function of PDLSCs remain unexplored.

Multiple active molecules, particularly lncRNAs, were recently reported to be tightly related to many biological processes, particularly cell differentiation and mitochondrial activity.^24–27,29,30,63^ However, lncRNAs are highly species- and tissue-specific,^24^ and previous studies have merely investigated the physiological function of lncRNAs in a normal environment. Our study explored the role of lncRNAs in regulating mitochondrial function and cementogenesis of human PDLSCs in an inflammatory environment. A better understanding of lncRNA functions in mitochondrial activity and the related mechanisms in a diseased environment will reveal novel and unanticipated biological insights with the potential to advance our knowledge of normal physiology and disease.^64^ In this study, we identified *GACAT2* as a key lncRNA associated with the inflammation-compromised cementogenesis of PDLSCs. Further gain/loss-of-function studies showed that *GACAT2* knockdown caused mitochondrial dysfunction in PDLSCs and in turn led to impaired cementoblastic differentiation (Fig. 4 and S9); indeed, *GACAT2* overexpression reversed the inflammation-compromised mitochondrial function and cementoblastic differentiation of PDLSCs (Fig. 5). *GACAT2* (*HMlincRNA717*) was previously reported to be aberrantly expressed in gastric cancer tissues, gastric precancerous lesions and gastric cell lines, which indicated that *GACAT2* may be pivotal in the occurrence and progression of gastric carcinoma.^65–67^ Our data provide the first indication that *GACAT2* is a key regulator of mitochondrial function and may thus be targeted to reverse the cementogenesis of PDLSCs in an inflammatory environment.

Given that *GACAT2* has been previously developed as a potential biomarker in the diagnosis of gastric cancer,^65^ we tentatively investigated the clinical significance of *GACAT2* expression in gingival tissues for root cementum damage. As expected, lower *GACAT2* expression was observed in gingival tissues of teeth with periodontitis with damaged cementum than in those from healthy teeth (Fig. S9). The use of *GACAT2* as a biomarker for predicting cementum damage requires further study.

A key feature of lncRNAs is that a stoichiometric interaction with their target molecule(s) is needed for the regulatory effects of lncRNAs.^64^ Thus, for determination of how functional lncRNAs regulate mitochondrial function during the cementoblastic differentiation of cells, the screening and identification of the target(s) of lncRNAs are pivotal for establishing the plausibility of this molecular mechanism. It has been reported that lncRNAs can be located in the cytoplasm or nucleus and function in diverse ways, such as interacting with RNA-binding proteins or functioning as a “sponge” to sequester miRNAs.^26,41,45,46,68^ In the current study, *GACAT2* was also found to be localized in both the cytoplasm and the nucleus of PDLSCs (Fig. 6a, b), which indicated a possible mechanism through which *GACAT2* might exert its biological functions via RNA-protein interactions. Moreover, GSEA predicted that *GACAT2* expression was significantly related to the mitochondrial inner membrane-associated gene signatures of PDLSCs (Fig. 6c), which is consistent with the above conclusion that *GACAT2* could affect mitochondrial function. Furthermore, we screened and identified *GACAT2*-binding proteins to explain how *GACAT2* influences mitochondrial function during the cementoblastic differentiation of cells. Through ChIRP-MS, PRM and RIP assays, we confirmed that *GACAT2* could directly bind to PKM1/2 (Fig. 6d-n), a molecule related to OXPHOS and ROS levels in mitochondria.^47–49,52^

The M1 form (PKM1) and M2 form (PKM2) of PK are produced from the *PKM* gene by differential splicing, and PK can catalyze the last step of glycolysis and convert phosphoenolpyruvate to pyruvate.^47,49,50^ In particular, PKM2, one of the major forms of PK, has been found to regulate metabolic reprogramming by multiple pathways, such as changing its expression, activity, allosteric regulation, post-translational modification and translocation.^48,50^ In the present study, we also found that the expression of PKM (PKM1/2 and PKM2), PK activity and pyruvate production were changed by the overexpression/inhibition of *GACAT2* in PDLSCs incubated in an inflammatory or noninflammatory environment (Fig. 7a-h). Because PKM2 tetramers are more enzymatically active than PKM2 monomers and dimers, the mechanism of higher PK activity could be a shift from PKM2 dimers or monomers to PKM2 tetramers.^48–50^ Here, a DSS crosslinking study was performed to assess the status of the allosteric regulation of PKM2. Consistent with the enhanced PK activity, PKM2 tetramerization was elevated by *GACAT2* overexpression (Fig. 7i-l). Furthermore, p-PKM2, an indicator of monomer/dimer formation,^50^ was increased or decreased in response to *GACAT2* overexpression or knockdown, respectively (Fig. 7m-p). Additionally, subcellular fractionation analysis and immunofluorescence staining for mitochondria and PKM2 in PDLSCs showed that *GACAT2* overexpression might result in the translocation of PKM2 into the mitochondria (Fig. 7q-s), and the translocation of PKM2 into mitochondria is a key step in PKM2 function.^51,52^ For example, mitochondrial PKM2 increases mitochondrial permeability and thereby generates more ATP for cell survival under glucose starvation.^51^ Although many studies have highlighted the essential role of mitochondrial PKM2 (such as ROS adaptation and ATP production), recent evidence shows that PKM2 may also translocate to the nucleus and then regulate glycolytic metabolism.^32^ The translocation of PKM2 to other organelles or nuclei to influence cellular events remains to be further explored.

Taken together, our functional studies demonstrated that *GACAT2* overexpression increased the cellular protein expression of PKM1/2, the PKM2 tetramer and p-PKM2 and led to enhanced PK activity along with increased translocation of PKM2 into the mitochondria (Fig. 7), which indicated that the interaction between *GACAT2* and PKM1/2 is key for regulating mitochondrial function and cementoblastic differentiation of PDLSCs in an inflammatory environment. PKM2 was previously reported to be a key metabolic regulator that affects cell differentiation by regulating mitochondrial fusion and fission,^50,69^ and some evidence has demonstrated that the activation of PKM2 may improve mitochondrial function by increasing mitochondrial metabolism.^48,70^ All of these findings indicate the crucial role of PKM2 in regulating mitochondrial function. In this study, we ultimately found that *GACAT2* overexpression could reverse the inflammation-compromised mitochondrial function and cementoblastic differentiation of PDLSCs, but this effect could be abolished by PKM1/2 knockdown (Fig. 8a-h). The data also demonstrate that *GACAT2* functions by binding to PKM1/2 to affect mitochondrial function and thereby regulate the cementoblastic differentiation of cells.

Despite recent advances in stem cell biology, the application of stem cell therapy in the management of many chronic inflammatory disorders, including periodontitis, remains in its infancy, largely due to the dysregulation of the reparative cell population exposed to inflammatory cues.^14–16,71^ Our data demonstrate for the first time a pivotal role of the lncRNA *GACAT2* in regulating the mitochondrial function and cementogenesis of PDLSCs in an inflammatory environment (Fig. 8i). We found that the overexpression of *GACAT2*, by binding to PKM1/2, could reverse the inflammation-compromised mitochondrial function and cementoblastic differentiation of PDLSCs. Our findings provide new insights into the cellular and molecular events that occur in inflammation-mediated cell differentiation and identify a new therapeutic target for promoting multiple tissue regeneration within a diseased periodontium, including the cementum.

## Materials and methods

Detailed materials and methods are provided in the Supplementary Information.

## Supplementary information

Supplementary Information

## Data Availability

The lncRNA microarray data are available in the GEO databases (accession number GSE176312).

## References

[1] Hajishengallis G, Chavakis T (2021). Local and systemic mechanisms linking periodontal disease and inflammatory comorbidities. Nat. Rev. Immunol..

[2] Kinane DF, Stathopoulou PG, Papapanou PN (2017). Periodontal diseases. Nat. Rev. Dis. Prim..

[3] Bartold PM, Gronthos S, Ivanovski S, Fisher A, Hutmacher DW (2016). Tissue engineered periodontal products. J. Periodontal Res..

[4] Xu XY (2019). Concise review: periodontal tissue regeneration using stem cells: strategies and translational considerations. Stem Cells Transl. Med..

[5] Chen FM, Jin Y (2010). Periodontal tissue engineering and regeneration: current approaches and expanding opportunities. Tissue Eng. Part B Rev..

[6] Nuñez J, Vignoletti F, Caffesse RG, Sanz M (2019). Cellular therapy in periodontal regeneration. Periodontol 2000.

[7] Arzate H, Zeichner-David M, Mercado-Celis G (2015). Cementum proteins: role in cementogenesis, biomineralization, periodontium formation and regeneration. Periodontol 2000.

[8] Nanci A, Bosshardt DD (2006). Structure of periodontal tissues in health and disease. Periodontol 2000.

[9] Li X (2019). M2 macrophages enhance the cementoblastic differentiation of periodontal ligament stem cells via the Akt and JNK pathways. Stem Cells.

[10] Seo BM (2004). Investigation of multipotent postnatal stem cells from human periodontal ligament. Lancet.

[11] El-Sayed KMF (2019). The periodontal stem/progenitor cell inflammatory-regenerative cross talk: a new perspective. J. Periodontal Res..

[12] Wang X (2017). MicroRNA-155-3p mediates TNF-α-inhibited cementoblast differentiation. J. Dent. Res..

[13] Xu XY (2020). Exosomes derived from P2X7 receptor gene-modified cells rescue inflammation-compromised periodontal ligament stem cells from dysfunction. Stem Cells Transl. Med..

[14] Yang H (2013). Comparison of mesenchymal stem cells derived from gingival tissue and periodontal ligament in different incubation conditions. Biomaterials.

[15] Lacey DC, Simmons PJ, Graves SE, Hamilton JA (2009). Proinflammatory cytokines inhibit osteogenic differentiation from stem cells: implications for bone repair during inflammation. Osteoarthr. Cartil..

[16] Xu XY (2019). Role of the P2X7 receptor in inflammation-mediated changes in the osteogenesis of periodontal ligament stem cells. Cell Death Dis..

[17] Wang Y (2020). IL1β inhibits differentiation of cementoblasts via microRNA-325-3p. J. Cell. Biochem..

[18] Li Q, Gao Z, Chen Y, Guan MX (2017). The role of mitochondria in osteogenic, adipogenic and chondrogenic differentiation of mesenchymal stem cells. Protein Cell.

[19] Bullon P, Newman HN, Battino M (2014). Obesity, diabetes mellitus, atherosclerosis and chronic periodontitis: a shared pathology via oxidative stress and mitochondrial dysfunction?. Periodontol 2000.

[20] De Mello AH, Costa AB, Engel JDG, Rezin GT (2018). Mitochondrial dysfunction in obesity. Life Sci..

[21] Van Horssen J, Van Schaik P, Witte M (2019). Inflammation and mitochondrial dysfunction: a vicious circle in neurodegenerative disorders?. Neurosci. Lett..

[22] Gjorgjieva T (2020). Loss of β‐actin leads to accelerated mineralization and dysregulation of osteoblast‐differentiation genes during osteogenic reprogramming. Adv. Sci..

[23] Li Y, Wang X (2016). Role of long noncoding RNAs in malignant disease. Mol. Med. Rep..

[24] Mirzadeh Azad F, Polignano IL, Proserpio V, Oliviero S (2021). Long noncoding RNAs in human stemness and differentiation. Trends Cell. Biol..

[25] Guo B, Zhu X, Li X, Yuan CF (2021). The roles of LncRNAs in osteogenesis, adipogenesis and osteoporosis. Curr. Pharm. Des..

[26] Yuan H (2019). A novel long noncoding RNA PGC1β-OT1 regulates adipocyte and osteoblast differentiation through antagonizing miR-148a-3p. Cell Death Differ..

[27] Yang L (2019). The long non-coding RNA-ORLNC1 regulates bone mass by directing mesenchymal stem cell fate. Mol. Ther..

[28] Dorn GW (2014). LIPCAR: a mitochondrial lnc in the noncoding RNA chain?. Circ. Res..

[29] Alessio E (2019). Single cell analysis reveals the involvement of the long non-coding RNA Pvt1 in the modulation of muscle atrophy and mitochondrial network. Nucleic Acids Res..

[30] Long J (2016). Long noncoding RNA Tug1 regulates mitochondrial bioenergetics in diabetic nephropathy. J. Clin. Invest.

[31] Zhang X (2019). Mechanisms and functions of long non-coding RNAs at multiple regulatory levels. Int. J. Mol. Sci..

[32] Hua Q (2020). Hypoxia-induced lncRNA-AC020978 promotes proliferation and glycolytic metabolism of non-small cell lung cancer by regulating PKM2/HIF-1α axis. Theranostics.

[33] Chowdhury AR (2020). Mitochondria-targeted paraquat and metformin mediate ROS production to induce multiple pathways of retrograde signaling: a dose-dependent phenomenon. Redox Biol..

[34] Zhao Q (2020). Targeting mitochondria-located circRNA SCAR alleviates NASH via reducing mROS output. Cell.

[35] Chen CT, Shih YR, Kuo TK, Lee OK, Wei YH (2008). Coordinated changes of mitochondrial biogenesis and antioxidant enzymes during osteogenic differentiation of human mesenchymal stem cells. Stem Cells.

[36] Pernas L, Scorrano L (2016). Mito-morphosis: mitochondrial fusion, fission, and cristae remodeling as key mediators of cellular function. Annu. Rev. Physiol..

[37] Bergman O, Ben-Shachar D (2016). Mitochondrial oxidative phosphorylation system (OXPHOS) deficits in schizophrenia: possible interactions with cellular processes. Can. J. Psychiatry.

[38] Wang CH, Wang CC, Huang HC, Wei YH (2013). Mitochondrial dysfunction leads to impairment of insulin sensitivity and adiponectin secretion in adipocytes. FEBS J..

[39] Song Y (2018). Sirtuin 3-dependent mitochondrial redox homeostasis protects against AGEs-induced intervertebral disc degeneration. Redox Biol..

[40] Lee YJ (2011). Suppression of human prostate cancer PC-3 cell growth by N-acetylcysteine involves over-expression of Cyr61. Toxicol. Vitr..

[41] Sun M, Kraus WL (2015). From discovery to function: the expanding roles of long noncoding RNAs in physiology and disease. Endocr. Rev..

[42] Feng S, Liang Y, Du W, Lv W, Li Y (2020). LncLocation: efficient subcellular location prediction of long non-coding RNA-based multi-source heterogeneous feature fusion. Int. J. Mol. Sci..

[43] Huang, Y. et al. Large scale RNA-binding proteins/LncRNAs interaction analysis to uncover lncRNA nuclear localization mechanisms. *Brief. Bioinform*. **22**, bbab195 (2021). 10.1093/bib/bbab195 34056657

[44] Wu N (2021). LINC00941 promotes CRC metastasis through preventing SMAD4 protein degradation and activating the TGF-β/SMAD2/3 signaling pathway. Cell Death Differ..

[45] Zhu Y (2020). LINC00467 is up-regulated by TDG-mediated acetylation in non-small cell lung cancer and promotes tumor progression. Oncogene.

[46] Wang X (2020). Long noncoding RNA HCP5 participates in premature ovarian insufficiency by transcriptionally regulating MSH5 and DNA damage repair via YB1. Nucleic Acids Res..

[47] Li T (2019). PKM2 coordinates glycolysis with mitochondrial fusion and oxidative phosphorylation. Protein Cell.

[48] Qi W (2017). Pyruvate kinase M2 activation may protect against the progression of diabetic glomerular pathology and mitochondrial dysfunction. Nat. Med..

[49] Zhou Q (2019). Inhibiting neddylation modification alters mitochondrial morphology and reprograms energy metabolism in cancer cells. JCI Insight.

[50] Yuan Q (2020). Role of pyruvate kinase M2-mediated metabolic reprogramming during podocyte differentiation. Cell Death Dis..

[51] Qi H (2019). Succinylation-dependent mitochondrial translocation of PKM2 promotes cell survival in response to nutritional stress. Cell Death Dis..

[52] Liang J (2017). Mitochondrial PKM2 regulates oxidative stress-induced apoptosis by stabilizing Bcl2. Cell Res.

[53] Lindskog S, Blomlöf L (1992). Mineralized tissue-formation in periodontal wound healing. J. Clin. Periodontol..

[54] Saygin NE, Giannobile WV, Somerman MJ (2000). Molecular and cell biology of cementum. Periodontol 2000.

[55] Foster BL (2012). Central role of pyrophosphate in acellular cementum formation. PLoS One.

[56] Ao M (2017). Overlapping functions of bone sialoprotein and pyrophosphate regulators in directing cementogenesis. Bone.

[57] D’Errico JA (1997). Expression of bone associated markers by tooth root lining cells, in situ and in vitro. Bone.

[58] Nuñez J, Sanz M, Hoz-Rodríguez L, Zeichner-David M, Arzate H (2010). Human cementoblasts express enamel-associated molecules in vitro and in vivo. J. Periodontal Res.

[59] Graves DT, Cochran D (2003). The contribution of interleukin-1 and tumor necrosis factor to periodontal tissue destruction. J. Periodontol..

[60] Delima AJ (2001). Soluble antagonists to interleukin-1 (IL-1) and tumor necrosis factor (TNF) inhibits loss of tissue attachment in experimental periodontitis. J. Clin. Periodontol..

[61] Iguchi M, Hiroi M, Kanegae H, Ohmori Y (2018). Costimulation of murine osteoblasts with interferon-γ and tumor necrosis factor-α induces apoptosis through downregulation of Bcl-2 and release of cytochrome c from mitochondria. Mediators Inflamm..

[62] López-Armada MJ (2006). Mitochondrial activity is modulated by TNFalpha and IL-1beta in normal human chondrocyte cells. Osteoarthr. Cartil..

[63] Wang Z (2021). A nuclear long non-coding RNA LINC00618 accelerates ferroptosis in a manner dependent upon apoptosis. Mol. Ther..

[64] Kopp F, Mendell JT (2018). Functional classification and experimental dissection of long noncoding rnas. Cell.

[65] Tan L, Yang Y, Shao Y, Zhang H, Guo J (2016). Plasma lncRNA-GACAT2 is a valuable marker for the screening of gastric cancer. Oncol. Lett..

[66] Shao Y (2014). Low expression of lncRNA-HMlincRNA717 in human gastric cancer and its clinical significances. Tumour Biol..

[67] Chen S, Li P, Xiao B, Guo J (2014). Long noncoding RNA HMlincRNA717 and AC130710 have been officially named as gastric cancer associated transcript 2 (GACAT2) and GACAT3, respectively. Tumour Biol..

[68] Han M (2020). lncRNA ZNF649-AS1 induces trastuzumab resistance by promoting ATG5 expression and autophagy. Mol. Ther..

[69] Guo J (2020). PKM2 suppresses osteogenesis and facilitates adipogenesis by regulating β-catenin signaling and mitochondrial fusion and fission. Aging.

[70] Stone OA (2018). Loss of pyruvate kinase M2 limits growth and triggers innate immune signaling in endothelial cells. Nat. Commun..

[71] Omoto M (2017). Hepatocyte growth factor suppresses inflammation and promotes epithelium repair in corneal injury. Mol. Ther..

