# Supplementary material for: LncRNA GACAT2 binds with protein PKM1/2 to regulate cell mitochondrial function and cementogenesis in an inflammatory environment
Source: Bone Res. 2022 Mar 16;10:29. doi: 10.1038/s41413-022-00197-x (PMC8927299; doi:10.1038/s41413-022-00197-x)
Supplement: Supplementary file 1 — Supplementary Information [file 41413_2022_197_MOESM1_ESM.pdf]

Supplementary Information

LncRNA *GACAT2* Binds with Protein PKM1/2 to Regulate Cell Mitochondrial Function and Cementogenesis in an Inflammatory Environment

Xuan Li, Bei-Min Tian, Dao-Kun Deng, Fen Liu, Huan Zhou, De-Qin Kong, Hong-Lei Qu, Li-Juan Sun, Xiao-Tao He,* Fa-Ming Chen*

Supplementary Information Includes the Following:

SUPPLEMENTARY MATERIALS AND METHODS

SUPPLEMENTARY FIGURES (16)

SUPPLEMENTARY TABLES (4)

Supplementary Materials and Methods

Isolation and Characterization of PDLSCs

Human healthy and intact permanent teeth (7 donors; aged 18 to 30 years) were used for the isolation of PDLSCs as previously described.[1] All donors agreed to provide signed informed consent and provided their extracted teeth for research purposes. Briefly, PDL tissue pieces were scraped from a washed tooth and treated with 3 mg/mL type I collagenase (DIYIBio; Shanghai, China). The tissue pieces were then incubated in α-MEM (Gibco BRL, Grand Island, NY, USA) containing 10% fetal bovine serum (FBS; Hangzhou Sijiqing Biological Engineering Materials, Zhejiang, China), 100 U/mL penicillin (Invitrogen, Carlsbad, CA, USA) and 100 mg/mL streptomycin (Invitrogen) until the primary cells migrated from the tissue pieces. Finally, the limiting dilution technique was used to obtain purified PDLSCs, and cells at passages 3-5 were used for subsequent studies. Their stemness and expansion capabilities were confirmed by colony-forming unit-fibroblast (CFU-F) assays, flow cytometric analysis, CCK-8 assay and analyses of their multiple differentiation potential as previously described.[1]

Cell Treatments

To induce cell cementoblastic differentiation, complete medium supplemented with 100 μg/mL EMD (Emdogain®, Straumann USA LLC, Andover, MA, USA) was used to establish cementoblastic conditions,[1] and the addition of 10 ng/mL TNF-α (Novoprotein Scientific Inc., Shanghai, China) plus 5 ng/mL IL-1β (Novoprotein Scientific Inc.) into the cell culture media was used to establish an inflammatory environment[2] for the identification of how the presence of inflammatory cytokines influences the cementoblastic differentiation of cells. To induce

mitochondrial dysfunction, cells were preincubated with an ROS generator (10 μM H_2O_2, Sigma-Aldrich, St. Louis, MO, USA) or mitochondrial inhibitor (10 μg/mL Oligo; TargetMol, Boston, MA, USA) for 24 h. To reverse the impaired mitochondrial function, the cells were preincubated with an ROS scavenger (1 mM NAC; Abcam, Cambridge, MA, USA; ab143032) or mitochondrial antioxidant (20 nM Visomitin; MedChemExpress) for 2 h.

Quantitative Real-Time Polymerase Chain Reaction (qRT-PCR)

qRT-PCR tests were conducted as previously described[1,3] with slight modifications. Briefly, total RNA was extracted using RNAiso Plus (Takara, Tokyo, Japan), and an aliquot of 500 ng of RNA was then reverse transcribed using Hifair® Ⅲ 1st Strand cDNA Synthesis SuperMix (Yeasen Biotech Company, Shanghai, China). The cDNA sample was subjected to qRT-PCR using SYBR Green Master Mix (Yeasen Biotech Company) and the CFX96 Real-Time PCR System (Bio-Rad, Hercules, CA, USA). The gene expression levels were normalized using *β-actin* (for mRNA or cytoplasm), *U6* (for nucleus) or *MTCO2* (for mitochondria). The following primers were used:

BSP (Forward), 5'-GAACCACTTCCCCACCTTTT-3';

BSP (Reverse), 5'-ATTCTGACCATCATAGCCATCG-3';

CEMP-1 (Forward), 5'-CTGAGGATGGGCACATCAAG-3';

CEMP-1 (Reverse), 5'-GAGGTTCTCAGCCGATGTGTTAG-3';

CAP (Forward), 5'-CGCGTGGACTGTGACAGAGA-3';

CAP (Reverse), 5'-GGCAAGGCAGCGTATATTGTAAGA-3';

β-actin (Forward), 5'-TGGCACCCAGCACAATGAA-3';

β-actin (Reverse), 5'-CTAAGTCATAGTCCGCCTAGAAGCA-3';

U6 (Forward), 5'-TGCTTCGGCAGCACATATAC-3';

U6 (Reverse), 5'-TCACGAATTTGCGTGTCATC-3';

MTCO2 (Forward), 5'-CTGCGACTCCTTGACGTTGAC-3';

MTCO2 (Reverse), 5'-GTAGCGGTGAAAGTGGTTTGGT-3';

PKM1/2 (Forward), 5'-CTGTGGCTGGACTACAAGAA-3';

PKM1/2 (Reverse), 5'-CTGCTTCACCTGGAGAGAAATA-3';

GACAT2 (Forward), 5'-CCCATTTCCTCCTGCTGTCT-3';

GACAT2 (Reverse), 5'-GCTGCTATGGAACCTTCTGTAA-3';

LINC01133 (Forward), 5'-AAACCTTTGCTCCAACTTTCTC-3';

LINC01133 (Reverse), 5'-CTTTGTTAATGGCTCCTCCAGT-3';

AC010247.2 (Forward), 5'-TCTGCTTCCGAGGCATTCA-3';

AC010247.2 (Reverse), 5'-CCTTCCCTTCTGTCCTTCCA-3';

AC096773.1 (Forward), 5'-ATGGGCACATTGTCATCAGG-3';

AC096773.1 (Reverse), 5'-TCATTTGGACCTACAGAACAGCA-3';

AL390957.1 (Forward), 5'-TGGTGAGGATGGCAGAGTAG-3';

AL390957.1 (Reverse), 5'-GCTTCTTAAATTCATCAGGGAA-3';

LINC01638 (Forward), 5'-GGAATCACACTGTCTCCTGTCA-3'; and

LINC01638 (Reverse), 5'-CATCCATCGCTCCTGTCTCT-3'.

AGTRAP (Forward), 5'-ATGTTTCTGGGTGGCTTGCT-3';

AGTRAP (Reverse), 5'-AGACCCAAGGAAACCAGTGT-3';

ZNF672 (Forward), 5'-CAGACTTGCAGGCTCACGG-3';

ZNF672 (Reverse), 5'-GCCGCAAAAACTGGTCTGAT-3';

GNAI2 (Forward), 5'-TTGCTGCTGTTGGGTGCTG-3';

GNAI2 (Reverse), 5'-TGGACTGGATGGTGTTGCTG-3';

C16orf71 (Forward), 5'-TGAGAGGGAGAGTGCTGAAGAA-3';

C16orf71 (Reverse), 5'-GAGGGTGCCATGCCTTTATC-3';

KIFC3 (Forward), 5'-ACGTACACGATGGAGGGGA-3';

KIFC3 (Reverse), 5'-CACAGCCGGATCTCCAGTTT-3';

CC2D1A (Forward), 5'-CGCATCGTCAAGCAATACCA-3';

CC2D1A (Reverse), 5'-CTTCATCCTCTGGGCCTTCA-3';

BARHL1 (Forward), 5'-GCCTGAAAAAGCCACGCAA-3';

BARHL1 (Reverse), 5'-GCTTCCATTTAGTCCTGCGGT-3';

TUBA8 (Forward), 5'-CAGGCCCAGCTGAGAGGT-3';

TUBA8 (Reverse), 5'-CACATGCTTCCCATTGCCAG-3';

CALML3 (Forward), 5'-TGCTCCCTGGTTGTGAAGTG-3';

CALML3 (Reverse), 5'-GCAGGACTAACGGCTCTGAA-3';

CMTM7 (Forward), 5'-TGGTAGCCGGAGCGATCTTTG-3';

CMTM7 (Reverse), 5'-GCATCTGTGGACTGGGTTACA-3';

Western Blot Analysis

Western blotting was conducted as previously described[4] with slight modifications. Briefly, the proteins were extracted and incubated overnight with the following primary antibodies: antibodies targeting BSP II (1:200, Santa Cruz Biotechnology, CA, USA; sc-73634), CEMP-1 (1:500, Abcam; ab134231) and CAP (3G9) (1:200, Santa Cruz Biotechnology; sc-53947) were used for cementoblastic samples; antibodies targeting NDUFB8 (1:1000, Proteintech, Wuhan, China; 14794-1-AP), SDHA (1:1000, Proteintech; 14865-1-AP), UQCRC1 (1:1000, Proteintech;

21705-1-AP), COXIV (1:5000, Proteintech; 11242-1-AP) and ATP5A (1:2000, Proteintech; 14676-1-AP) were used for mitochondrial respiratory chain complex analysis; and antibodies targeting PKM1/2 (1:1000, Cell Signaling Technology; #3106), PKM1 (1:1000, Cell Signaling Technology; #7067), PKM2 (1:1000, Cell Signaling Technology; #4053) and phospho-PKM2 (Tyr105; 1:1000, Cell Signaling Technology; #3827) were used for PKM analysis. The protein expression levels were normalized using β-actin (1:5000, Proteintech; 20536-1-AP). Protein quantification was conducted using the relative gray values of blots.

Alkaline Phosphatase (ALP) Activity Assay

Culture supernatants of PDLSCs were collected for ALP activity assays using an ALP activity detection kit (Jiancheng Bioengineering, Nanjing, China). The cell samples were fixed for ALP staining using a BCIP/NBT Alkaline Phosphatase Color Development Kit (Beyotime, Haimen, China).

Determination of Mitochondrial Function

ROS Levels

According to a previous study,[4,5] the levels of cellular ROS and mtROS in PDLSCs were determined using dihydroethidium (DHE, Beyotime) and MitoSOX (Invitrogen, Carlsbad, CA, USA), respectively. PDLSCs were collected and cultivated in medium loaded with 5 μM DHE or 5 μM MitoSOX at 37 °C in the dark for 30 min. The cells were rinsed twice with phosphate buffered saline (PBS), and the relative fluorescence intensity of DHE and MitoSOX was recorded using a flow cytometer (BD Accuri C6, San Jose, CA, USA). The excitation

wavelength (EX)/emission wavelength (EM) values of DHE and MitoSOX were 535/610 nm and 510/580 nm, respectively.

Mitochondrial Membrane Potential (MMP)

The MMP of PDLSCs was investigated using a JC-1 MMP Assay Kit (G-CLONE, Beijing, China). Briefly, a sample of 5×10^4 PDLSCs was seeded in each confocal dish. After the different treatments, the cells were rinsed twice with PBS and loaded with JC-1 staining working solution at 37 °C for 20 min. The confocal dishes (washed with JC-1 dilution buffer) were immediately observed with a fluorescence microscope (BX51; Olympus Optical, Tokyo, Japan). At least 3 microscopic fields were randomly selected from each independent experiment, and the fluorescence intensity was quantified using ImageJ software (NIH ImageJ, Bethesda, MD, USA). The relative red/green fluorescence ratio was acquired as an index of the MMP (green, EX/EM: 490/530 nm; red, EX/EM: 525/590 nm).

Intracellular Adenosine 5′-triphosphate (ATP) Content

The intracellular ATP content of PDLSCs was investigated using a commercially available ATP assay kit (Beyotime). Briefly, 1×10^6 PDLSCs were lysed and centrifuged at 4 °C. After removal of the cell pellets, the supernatant (20 μL each well) was transferred to black 96-well plates (Thermo Scientific, Waltham, MA, USA) containing ATP working solution (100 μL each well). The luminescence was then recorded with a microplate reader (Infinite M200 Pro, Tecan, Switzerland), and the ATP concentration was further divided by the protein concentration of cells detected using the bicinchoninic acid assay (Beyotime).

Mitochondrial DNA (mtDNA) Content

To determine the mtDNA content of PDLSCs,[6] DNA was first isolated from PDLSCs using the QIAamp DNA Mini Kit (Qiagen, Valencia, CA, USA), and DNA was then subjected to RT-PCR using the PCR detection kit (TSINGKE, Beijing, China; TSE301) and CFX96 Real-Time PCR System (Bio-Rad) with the aid of specific primers and probes (FAM-labeled TAMRA-quenched probes) for the *NADH dehydrogenase subunit 1* (*ND1*) gene (mtDNA) and *β-actin* (nuclear DNA, nDNA). The mtDNA amplification signals were normalized to the nDNA signal (mtDNA/nDNA ratio). The following primers and probes were used in the assay:

β-actin (Forward), 5'-GACTACCTCATGAAGATCCTCACC-3';

β-actin (Reverse), 5'-TCTCCTTAATGTCACGCACGATT-3';

β-actin (Probe), 5'-(FAM) CGGCTACAGCTTCACCACCACGGC (Eclipse)-3';

ND1 (Forward), 5'-CCCTTATCACAACACAAG-3';

ND1 (Reverse), 5'-TCGGCTATGAAGAATAGG-3'; and

ND1 (Probe), 5'-(FAM) ACACCTCTGATTACTCCTGCCATC (Eclipse)-3'.

Transmission Electron Microscopy (TEM)

A sample of 1×10^7 PDLSCs was collected and processed as described previously.[4] Briefly, the PDLSCs were fixed in 4% glutaraldehyde overnight at 4 °C, rinsed, postfixed with 1% osmium tetroxide, dehydrated using a gradient of ethanol, and embedded in resin. Ultrathin sections were then prepared and double stained using uranyl acetate and lead citrate. The ultrastructures of the cells were viewed under a Tecnai G2 transmission electron microscope (Tecnai G2 Spirit BioTWIN; FEI Company, Hillsboro, OR, USA).

Oxygen Consumption Rate (OCR)

The OCR of the PDLSCs was measured using Seahorse XF24 equipment (Agilent Technologies, Santa Clara, CA, USA) as previously described[4] with slight modifications. Briefly, 1×10^4 PDLSCs were seeded in each well of the microplate, and the cells were incubated overnight in 200 μl of medium under different conditions. The microplates were then equilibrated without CO_2 for 1 h prior to measurement. Subsequently, the ATPase inhibitor oligomycin (Oligo, 1 μM), the uncoupling reagent carbonyl cyanide-p-trifluoromethoxyphenylhydrazone (FCCP, 1 μM), and inhibitors of the electron transport chain rotenone/antimycin (R/A, 2 μM) were sequentially injected during real-time measurements of the OCR, whereas respiratory parameters were measured after each injection. Cellular Respiration: basic OCR value prior to the Oligo injection; Maximal Respiration: differences between the maximum rate measurement after FCCP injection and the nonmitochondrial respiration (minimum rate measurement after the R/A injection); ATP Production: difference between the final rate measurement prior to the Oligo injection and the minimum rate measurement after the Oligo injection.

lncRNA Microarray Analysis

Total RNA of PDLSCs incubated in a noninflammatory environment (EMD group) and an inflammatory environment (Infla-EMD group) (n = 3) was extracted using TRIzol Reagent (Invitrogen). The samples were then amplified and transcribed to fluorescent cRNAs, and the labeled cRNAs were subjected to a lncRNA microarray using an Arraystar Human lncRNA Array (ArrayStar, Rockville, MD, USA) according to previous studies.[7,8] Microarray scanning and data analysis were performed by KangChen Biotech (China). The lncRNA microarray data are available in the GEO databases (accession number GSE176312). The differentially expressed

lncRNAs between the EMD and Infla-EMD groups were screened by volcano plot filtering, and the top 8 upregulated and top 8 downregulated lncRNAs were visualized using a heatmap. Information on the 16 screened lncRNAs is listed in Table S1. The potential lncRNAs were further screened according to their RNA length (< 2,000 nt), intensity (>100), relation (without overlapping with coding transcripts) and database source (in GENCODE or RefSeq public databases). qRT-PCR was then performed to validate the abovementioned differentially expressed lncRNAs.

Transfections

For the silencing of target lncRNAs, small interfering RNAs (siRNAs) targeting potential lncRNAs and a negative control (si-NC) were purchased from TSINGKE (Beijing, China). The sequences of the siRNAs were as follows: si-*GACAT2*, 5'-CCAACUCUAAGCUCAUCUUTT-3'; si-*LINC01133*, 5'-GGAGCCAUUAACAAAGCUUTT-3'; si-*AC010247.2*, 5'-GAGCUGCCUCUACACUAAATT-3'; si-*AC096773.1*, 5'-GUUAUCCAAAGUUAAGACATT-3'; si-PKM1/2, 5'-GCAGCAAGAUCUACGUGGATT-3'; and si-NC, 5'-UUCUCCGAACGUGUCACGUTT-3'. PDLSCs were transfected with si-NC or specific siRNA (20 μM) using Micropoly Transfecter™ Cell Reagent (Micropoly, Nantong, China) according to the manufacturer's recommended protocol. Briefly, 8 μL of the transfection reagent was first mixed with 8 μL of si-NC or specific siRNA (20 μM). The mixture was incubated at room temperature for 10 min, and 16 μL of the mixture was diluted in 1200 μL of culture medium and added to 6-well plates (5×10^4 PDLSCs). After 72 h, the silencing efficiency of each lncRNA in the PDLSCs was evaluated by qRT-PCR.

To overexpress the target lncRNA, the sequence of the target lncRNA was cloned into a lentiviral vector pLVX-IRES-Puro (TSINGKE). PDLSCs were then transfected with the abovementioned lentivirus according to the manufacturer's protocol. The nonspecific lentiviral vector served as a negative control vector (ov-NC). Briefly, after identifying a suitable multiplicity of infection, 2×10^5 PDLSCs in 6-well plates were transfected with the lentivirus (MOI = 10) overnight. The medium was then replaced with fresh complete α-MEM medium. After a selection procedure using 5 µg/mL puromycin (DIYIBio) for 48 h, surviving PDLSCs were collected and reseeded in 60-mm-diameter culture dishes (Thermo Fisher Scientific, Waltham, MA, USA). After 2-4 passages, the overexpression efficiency of the target lncRNA in the PDLSCs was evaluated by qRT-PCR.

Cytoplasm/Nucleus Fraction Isolation

The extraction of nuclear and cytoplasmic fractions from PDLSCs was carried out using a Minute™ Cytoplasmic and Nuclear Extraction Kit (Invent Biotechnologies, Plymouth, MN, USA) according to the manufacturer's instructions. The RNAs extracted from nuclear and cytoplasmic fractions were purified and reverse transcribed, and qRT-PCR was then performed using standard protocols. The extracted RNAs were identified using nuclear markers (*U6*) and cytoplasmic markers (*β-actin*). Moreover, the expression of lncRNAs along with nuclear (*U6*) and cytoplasmic references (*β-actin*) in each fraction was normalized to their levels in the RNA samples from whole cells, which was set to 100%.[3,8]

Mitochondrial Fraction Isolation

The extraction of mitochondrial and cytoplasmic fractions from PDLSCs was carried out using a Minute™ Mitochondria Isolation Kit (Invent Biotechnologies) according to the manufacturer's instructions. The RNAs and proteins extracted from mitochondrial and cytoplasmic fractions were detected by qRT-PCR and Western blot assays, respectively. The extracted RNAs were identified using mitochondrial markers (*MTCO2*) and cytoplasmic markers (*β-actin*). The extracted proteins were identified using mitochondrial markers (COXIV).

RNAscope® In Situ Hybridization (RNAscope)

To verify the subcellular localization of *GACAT2*, a custom *GACAT2* RNAscope probe for humans was developed prior to initiating this experiment, and this probe is currently listed in the Advanced Cell Diagnostics catalog as Hs-*GACAT2*, NR_120598.1, target region: 2–822 (cat. no. 902381). PDLSCs were seeded on a Lab-Tek II chamber slide system (Thermo Fisher Scientific; 154534) and cultured in Nor, EMD and Infla-EMD medium, and the lncRNA *GACAT2* of PDLSCs from each group was then pretreated and detected with an RNAscope® multiplex fluorescent reagent kit v2 (Advanced Cell Diagnostics, CA, USA; 323100) according to the manufacturer's protocols. Briefly, the cells were rinsed twice with PBS and fixed with 4% paraformaldehyde (Thermo Fisher Scientific) for 30 min. Subsequently, the cells were dehydrated, rehydrated, treated with hydrogen peroxide (Advanced Cell Diagnostics; 322381) and protease III (Advanced Cell Diagnostics; 322000), and washed with PBS. After the above-described pretreatment, the slides were placed in HybEZ™ Slide Rack. Negative control probes, positive control probes and *GACAT2* probes were then added to the PDLSCs for 2 h at 40 °C within a HybEZ™ humidity control tray. Following probe incubation, the cells were incubated sequentially with AMP 1 for 30 min, AMP 2 for 30 min, AMP 3 for 15 min, HRP-C1 for 15 min,

Opal 520 (PerkinElmer, Waltham, MA; ASOP520) for 30 min and HRP Blocker for 15 min at 40 °C. Before the addition of each of the abovementioned reagents, the PDLSCs were rinsed twice with washing buffer (Advanced Cell Diagnostics, 310091). The PDLSCs were then counterstained with DAPI for 30 sec and mounted using Prolong Gold. Images were captured using a Nikon confocal laser microscope (Nikon, A1 PLUS, Tokyo, Japan).

Gene Set Enrichment Analysis (GSEA)

GSEA based on the correlation between *GACAT2* and mRNAs was performed using GSEA software v2.2.0. Gene sets that met the criteria false discovery rate (FDR) < 0.25 and $p < 0.05$ were selected.

Construction of ceRNA network

The ceRNA network was constructed by miRNA target prediction software (based on TargetScan & miRanda) as previously described[9] with slight modifications.

Comprehensive Identification of RNA-Binding Proteins by Mass Spectrometry (ChIRP-MS)

ChIRP assays were conducted using *GACAT2*-specific biotin probes as previously described[10,11] with slight modifications. Briefly, 1×10^8 PDLSCs were resuspended in prechilled PBS and crosslinked with 3% formaldehyde (Sangon Biotech, Shanghai, China). After quenching the crosslinking with 125 mM glycine, the PDLSCs were sonicated in lysis buffer. Prebinding probes of the control and *GACAT2* were then added to streptavidin beads and used overnight hybridization with the cell lysates. The following day, the beads were rinsed with wash buffer, and 1/20 of the beads were reserved for qRT-PCR. The remaining 19/20 beads were resuspended

in 100 μL of elution buffer containing benzonase (20 U) for 1 h at 37 °C to liberate the lncRNA-associated proteins. The supernatant was then transferred to a low-binding tube, and elution of beads was repeated followed by a combination of two supernatants. After the crosslinking of the sample was reversed at 95 °C, the protein was precipitated using 0.1% SDC and 10% TCA at 4 °C. The samples were centrifuged at top speed and washed three times with prechilled 80% acetone.

For liquid chromatography-tandem mass spectrometry (LC-MS/MS), tryptic digestion and peptide desalting of the samples were conducted, and 5 μL of peptide from each sample was separated and measured with a nano-UPLC (EASY-nLC1200) coupled to Q-Exactive MS (Thermo Finnigan). The spectral data were analyzed using MaxQuant Ver 1.6.1.0 and the protein sequence database (UniProt Database). U1 probes (positive control) retrieved the known direct binding protein U1A, whereas the nontargeting probes (negative control) did not bind human RNA. The proteins precipitated by *GACAT2* probes were quantified based on the fold change of normalized spectral counts relative to the negative control. The results of the ChIRP-MS assay are listed in Table S3. The ChIRP probe (*GACAT2*) sequences used are as follows:

2010031A-P1 5'-AGGGGAGTTGTATTGACACC-3';

2010031A-P2 5'-CATCTTTTCTCCACGGAGGG-3';

2010031A-P3 5'-TGCACTCTCTGAGTATTTTC-3';

2010031A-P4 5'-TCCTTTGTAAGCATCCAGGA-3';

2010031A-P5 5'-TTTTCGGTGAGCAAGCTGAA-3';

2010031A-P6 5'-TCAGGATAATGCCACTGGAG-3';

2010031A-P7 5'-AATAATGTTGCCAGATGCCA-3';

2010031A-P8 5'-TGGAAAGCCAGACTTCCTTC-3'; and

2010031A-P9 5'-CTGTTATTTGCTGCTATGGA-3'.

Parallel Reaction Monitoring (PRM)

PRM was performed using a nano-UPLC instrument (EASY-nLC1200) coupled to a Q-Exactive mass spectrometer (Thermo Finnigan) as previously described[11-13] with slight modifications. Tryptic peptides of each sample were dissolved in buffer A (0.1% formic acid in water) and separated by a 100-µm-ID × 15-cm reversed-phase chromatographic column. Subsequently, elution was performed over a 120-min gradient at a rate of 300 nL/min with buffer B (0.1% formic in 80% acetonitrile). After nano-UPLC separation, PRM data acquisition was conducted using LC-MS/MS. Finally, the collected PRM data were imported into Skyline software for transition extraction. For endogenous peptide quantification, all product ion peak areas were calculated, and the values were normalized to the controls (nontargeting probes). The criteria used to screen differentially expressed peptides were as follows: fold change > 1.5 and adjusted $p < 0.05$. The results of the PRM assay are listed in Table S4.

RNA Immunoprecipitation (RIP)

The RIP experiment was conducted using the Magna RIP kit (Millipore, Billerica, MA, USA; Cat# 17-700) as previously described[4,7,8] with slight modifications. Briefly, approximately 1×10^7 PDLSCs were lysed with RIP lysis buffer supplemented with protease inhibitor cocktail and RNase inhibitor. The lysates were then subjected to immunoprecipitation overnight at 4 °C with anti-PKM1/2 antibodies (1:50, Cell Signaling Technology, Danvers, MA, USA; #3106) and anti-IgG antibodies (isotype control). After immunoprecipitation, the coprecipitated RNAs were extracted and purified using the phenol-chloroform method, and the purified RNAs were reverse-

transcribed. The fold enrichment of the precipitated lncRNA *GACAT2* was then examined by qRT-PCR. The proteins isolated from the magnetic beads were detected by immunoblotting analysis.

Assay of Pyruvate Kinase (PK) Activity and Pyruvate Production

PK activity was evaluated using a PK activity assay kit (Solarbio, Beijing, China; BC0545). Pyruvate production was evaluated using a pyruvate assay kit (Jiancheng Bioengineering; A081). All values were normalized according to the protein concentration of cells.

Crosslinking Assay

The cells in each group were trypsinized and resuspended in PBS (pH 8.0). The suspended cells were crosslinked with 5 mM disuccinimidyl suberate (DSS, Thermo Fisher Scientific; A39267) for 30 min, and the crosslinking reaction was terminated with 20 mM Tris-HCl (pH 7.5, 1 M). Western blotting with the indicated antibody (rabbit anti-PKM2; 1:1000, Cell Signaling Technology; #4053) was then conducted.

Immunofluorescence Staining

Immunofluorescence labeling was conducted to investigate the relocation of PKM2. For mitochondrial staining, the cells were washed and then incubated with 100 nM MitoTracker® Red CMXRos (Invitrogen; M7512) for 30 min at 37 °C. The cell samples were then rinsed, fixed with 4% paraformaldehyde, and incubated with specific primary antibody (rabbit anti-PKM2; 1:100, Cell Signaling Technology; #4053) and secondary antibody (Alexa 488 AffiniPure Donkey Anti-Rabbit IgG; 1:50, Yeasen) in an appropriate diluent. The cells were subsequently

stained with DAPI (1:1000, Bioworld, Nanjing, China; BD5010) for 10 min in the dark. Images were observed with a Nikon confocal laser microscope (Nikon, A1 PLUS, Tokyo, Japan).

Animal Experiments

To evaluate how inflammation influences cell cementogenesis in vivo, PDLSCs were incubated within a normal or inflammatory environment (supplemented with EMD and 50 μg/mL vitamin C, Vc; Sigma-Aldrich; EMD or Infla-EMD group, respectively) to form cell sheets, and the obtained cell sheets combined with treated dentin matrix (TDM) were subcutaneously transplanted into male nude mice as previously described[14,15] with slight modifications. Briefly, PDLSCs were seeded on 60-mm-diameter culture dishes (Thermo Fisher Scientific) and cultured in medium with EMD plus Vc (EMD group) or EMD plus Vc and inflammatory cytokines (Infla-EMD) for 14 days to form cell sheets. Human TDM, the cell carrier, was fabricated as previously described.[14,15] To obtain human TDM, cleaned and rounded human dentins (with a diameter of approximately 5 mm and a thickness of 1 mm) derived from healthy and intact permanent teeth (extracted for impaction-related reasons) were treated with 17%, 10% and 5% ethylenediaminetetraacetic acid (EDTA; Sigma-Aldrich) and then maintained in sterile PBS with 100 U/mL penicillin (Invitrogen) and 100 mg/mL streptomycin (Invitrogen) for 72 h. Subsequently, TDM was sterilized with Co60 radiation and stored in α-MEM at 4 °C. The cell sheets of each group combined with TDM were subcutaneously transplanted into the dorsum of male nude mice (aged 6 weeks; FMMU, Animal Centre, Xi'an, China).

Similarly, to investigate whether *GACAT2* overexpression could reverse inflammation-compromised cell cementogenesis in vivo, PDLSCs that were incubated in an inflammatory environment (EMD plus Vc and inflammatory cytokines) and transfected with ov-*GACAT2* or

ov-NC were subcutaneously transplanted into nude mice as previously described.[14,15] The research protocol was approved by the Laboratory Animal Care & Welfare Committee, School of Stomatology, Fourth Military Medical University (FMMU; protocol number: 2020004). At 8 weeks postimplantation, the transplants were harvested and assessed by histological analysis.

Histological Analysis of Transplants

For immunofluorescence labeling, the transplants were fixed using 4% paraformaldehyde, decalcified in EDTA-decalcification solution (Coolaber, Beijing, China) for approximately 3 months, dehydrated with 30% sucrose and embedded in Tissue-Tek® O.C.T. Compound (Sakura Finetek USA, Torrance, CA, USA). The sections were cut into 15-µm-thick sections, incubated with anti-CEMP-1 (1:100, Abcam; ab134231) or anti-CAP (1:50, Abcam; ab192772) antibodies and stained with DAPI (1:1000, Bioworld, Nanjing, China; BD5010) for 10 min in the dark. Images were obtained with a fluorescence microscope (BX51; Olympus Optical, Tokyo, Japan).

For hematoxylin and eosin (H&E) staining, the transplants were fixed, decalcified and embedded in paraffin. The samples were cut into thin sections (5 µm) and then analyzed by HE staining. Finally, the sections were subjected to morphological observation using a microscope (BX50; Olympus Optical).

Clinical Specimens

Healthy teeth (extracted for impaction-related reasons) and destroyed teeth (extracted for periodontitis-related reasons) were collected from patients who underwent tooth extraction in the dental clinic of the FMMU. Gingival tissues were obtained from healthy or periodontitis teeth for histopathological assessment. All donors provided signed written informed consent and provided

their extracted teeth or tissues for our research purposes. The research protocol was conducted in accordance with the Declaration of Helsinki and approved by the review board of the School of Stomatology, FMMU.

Methylene Blue-Acid Fuchsin

Morphological observation of clinically collected teeth was conducted using methylene blue-acid fuchsin. Briefly, the samples were dehydrated and embedded in methyl methacrylate. The embedded teeth were then cut and ground into thin sections (20-40 μm) using a macrocutting system (Leica Microtome, Wetzlar, Germany) and micro grinding system (EXAKT, Hamburg, Germany). These sections were then stained with methylene blue and acid fuchsin (Shanghai Yuanye Bio-Technology Co., Ltd, Shanghai, China; R23038).

H&E Staining

The morphology of the tissue was observed based on H&E staining. The collected tissues were fixed using 4% paraformaldehyde and embedded in paraffin. The samples were cut into thin sections (5 μm) and then analyzed by HE staining. Finally, the sections were subjected to morphological observation using a microscope (BX50; Olympus Optical).

ROS Generation in Tissues

The cellular ROS and mtROS levels of collected tissues were determined using DHE (Beyotime) and MitoSOX probes (Invitrogen), respectively. Briefly, tissues were collected, rinsed and immediately embedded in Tissue-Tek® O.C.T. Compound (Sakura Finetek USA). The samples were cut into 8-μm-thick sections and cultivated in medium loaded with 5 μM DHE or 5 μM

MitoSOX at 37 °C in the dark for 30 min. Finally, images were obtained with a fluorescence microscope (BX51; Olympus Optical).

Statistical Analysis

Cells isolated from 7 donors were used in the present study. The statistical analyses were conducted using GraphPad Prism 7 software. When comparing more than two groups, the experimental data were analyzed by one-way analysis of variance (ANOVA) followed by Tukey's multiple comparisons tests, Sidak's multiple comparisons tests or Dunnett's multiple comparisons. Two-group comparisons were conducted using unpaired 2-tailed Student's *t* tests. The results are presented as the means ± SDs of at least 3 independent experiments. Statistical significance was indicated by $P < 0.05$ (*), $P < 0.01$ (**) or $P < 0.001$ (***).

References

1. Li, X. *et al.* M2 macrophages enhance the cementoblastic differentiation of periodontal ligament stem cells via the Akt and JNK pathways. *Stem Cells* **37,** 1567–1580 (2019).
2. Xu, X. Y. *et al.* Role of the P2X7 receptor in inflammation-mediated changes in the osteogenesis of periodontal ligament stem cells. *Cell Death Dis.* **10,** 20 (2019).
3. Kong, P. *et al.* Circ-Sirt1 controls NF-κB activation via sequence-specific interaction and enhancement of SIRT1 expression by binding to miR-132/212 in vascular smooth muscle cells. *Nucleic Acids Res.* **47,** 3580–3593 (2019).
4. Zhao, Q. *et al.* Targeting mitochondria-located circRNA SCAR alleviates NASH via reducing mROS output. *Cell* **183,** 76–93.e22 (2020).
5. Yuan, K. *et al.* HBV-induced ROS accumulation promotes hepatocarcinogenesis through Snail-mediated epigenetic silencing of SOCS3. *Cell Death Differ.* **23,** 616–627 (2016).
6. Chen, C. T., Shih, Y. R., Kuo, T. K., Lee, O. K. & Wei, Y. H. Coordinated changes of mitochondrial biogenesis and antioxidant enzymes during osteogenic differentiation of human mesenchymal stem cells. *Stem Cells* **26,** 960–968 (2008).
7. Huang, D. *et al.* NKILA lncRNA promotes tumor immune evasion by sensitizing T cells to activation-induced cell death. *Nat. Immunol.* **19,** 1112–1125 (2018).
8. Wang, X. *et al.* Long noncoding RNA HCP5 participates in premature ovarian insufficiency by transcriptionally regulating MSH5 and DNA damage repair via YB1. *Nucleic Acids Res.* **48,** 4480–4491 (2020).
9. Wan J, Liu B. Construction of lncRNA-related ceRNA regulatory network in diabetic subdermal endothelial cells. *Bioengineered*. **12**:2592-2602 (2021).

10. Chai, P. *et al.* Dynamic chromosomal tuning of a novel GAU1 lncing driver at chr12p13.32 accelerates tumorigenesis. *Nucleic Acids Res.* **46,** 6041–6056 (2018).

11. Zhu, Y. *et al.* LINC00467 is up-regulated by TDG-mediated acetylation in non-small cell lung cancer and promotes tumor progression. *Oncogene* **39,** 6071–6084 (2020).

12. Wingo, T. S. *et al.* Integrating next-generation genomic sequencing and mass spectrometry to estimate allele-specific protein abundance in human brain. *J. Proteome Res.* **16,** 3336–3347 (2017).

13. Lu, S. *et al.* A hidden human proteome encoded by 'non-coding' genes. *Nucleic Acids Res.* **47,** 8111–8125 (2019).

14. Yang, H. *et al.* Treated dentin matrix particles combined with dental follicle cell sheet stimulate periodontal regeneration. *Dent. Mater.* **35,** 1238–1253 (2019).

15. Yang, B. *et al.* Tooth root regeneration using dental follicle cell sheets in combination with a dentin matrix - based scaffold. *Biomaterials* **33,** 2449–2461 (2012).

Supplementary Figures

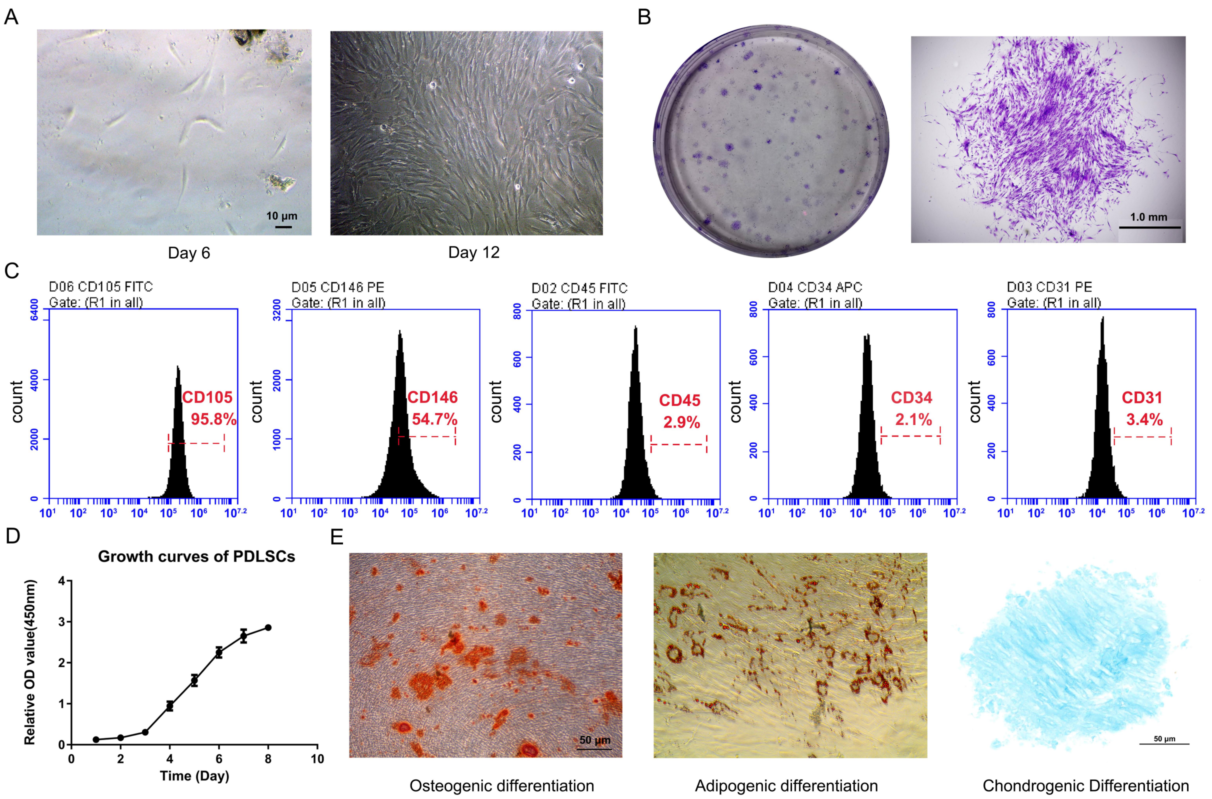

Fig. S1. Isolation and characterization of PDLSCs. (**a**) Primary cells migrated from PDL tissues observed on day 6 and day 12 (scale bar: 10 μm). (**b**) Cell colonies formed by PDLSCs observed in the dish (general view) and amplified image of a representative colony (scale bar: 1 mm). (**c**) Positive expression of CD105 and CD146 and negative expression of CD45, CD34 and CD31 in PDLSCs (flow cytometric analysis). (**d**) Growth curve of PDLSCs (CCK-8 assay) during a culture period of 8 days. (**e**) Multiple differentiation potentials of PDLSCs (from left to right: osteogenic differentiation, adipogenic differentiation and chondrogenic differentiation; scale bar: 50 μm).

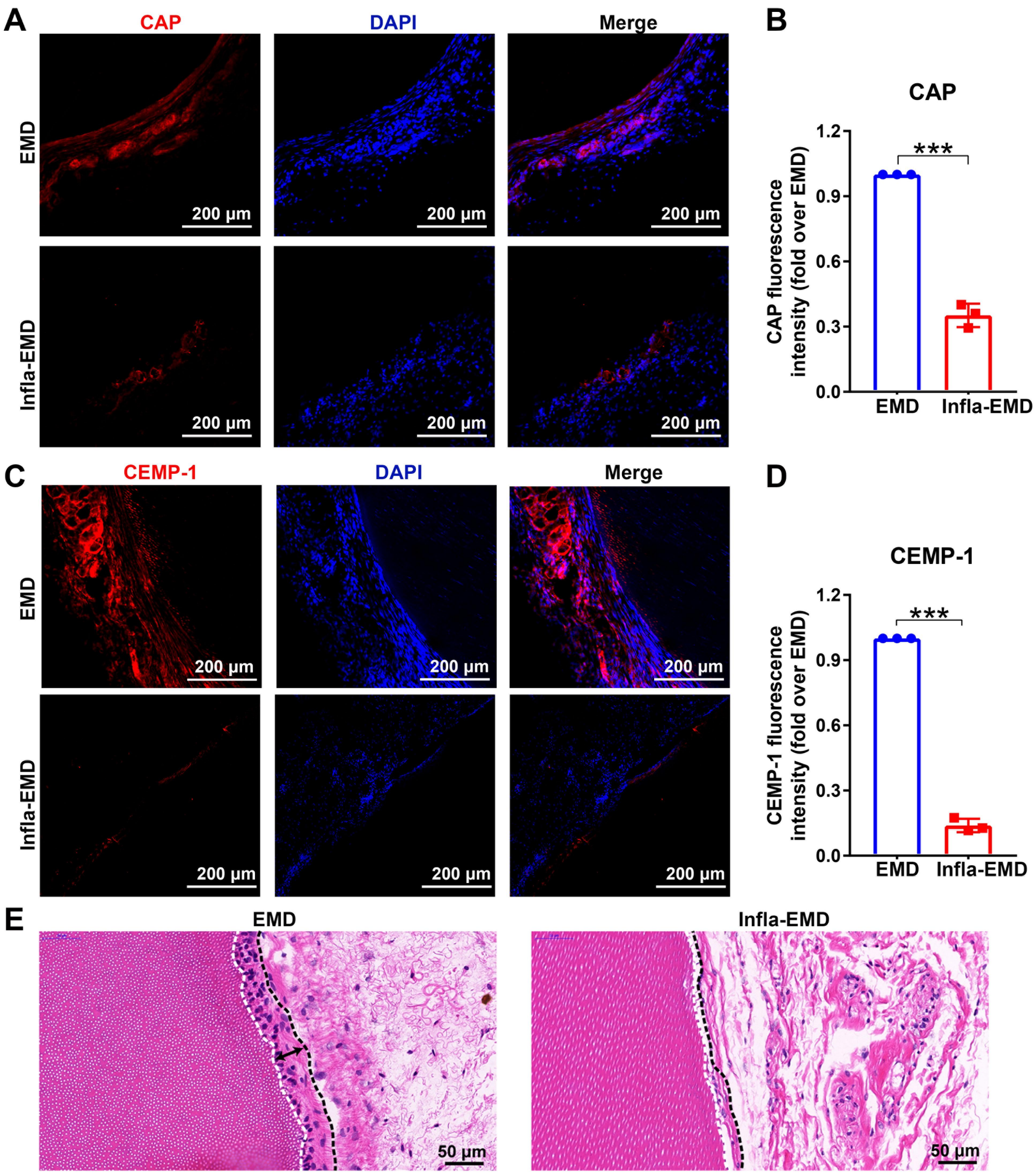

Fig. S2. Inflammatory cytokines compromise cementogenesis in vivo. The cells were incubated within normal or inflammatory conditions (supplemented with EMD and Vc; EMD or Infla-EMD group, respectively) to form cell sheets, and the obtained cell sheets combined with treated dentin matrix (TDM) were subcutaneously transplanted into nude mice. (**a**) CAP expression in transplants derived from the EMD or Infla-EMD group (immunofluorescence staining; scale bar: 200 μm). (**b**) Qualification analysis of CAP expression levels in transplants derived from the EMD or Infla-EMD group. (**c**) CEMP-1 expression in transplants derived from the EMD or

Infla-EMD group (immunofluorescence staining; scale bar: 200 μm). (**d**) Qualification analysis of CEMP-1 expression levels in transplants derived from the EMD or Infla-EMD group. (**e**) Histopathological assessment of transplants derived from the EMD or Infla-EMD group (H&E staining; arrows indicate cementum newly formed by PDLSC sheets; scale bar: 50 μm). The data are shown as the means ± SDs for $n = 3$; $^{***}P < 0.001$ indicate significant differences between the indicated columns.

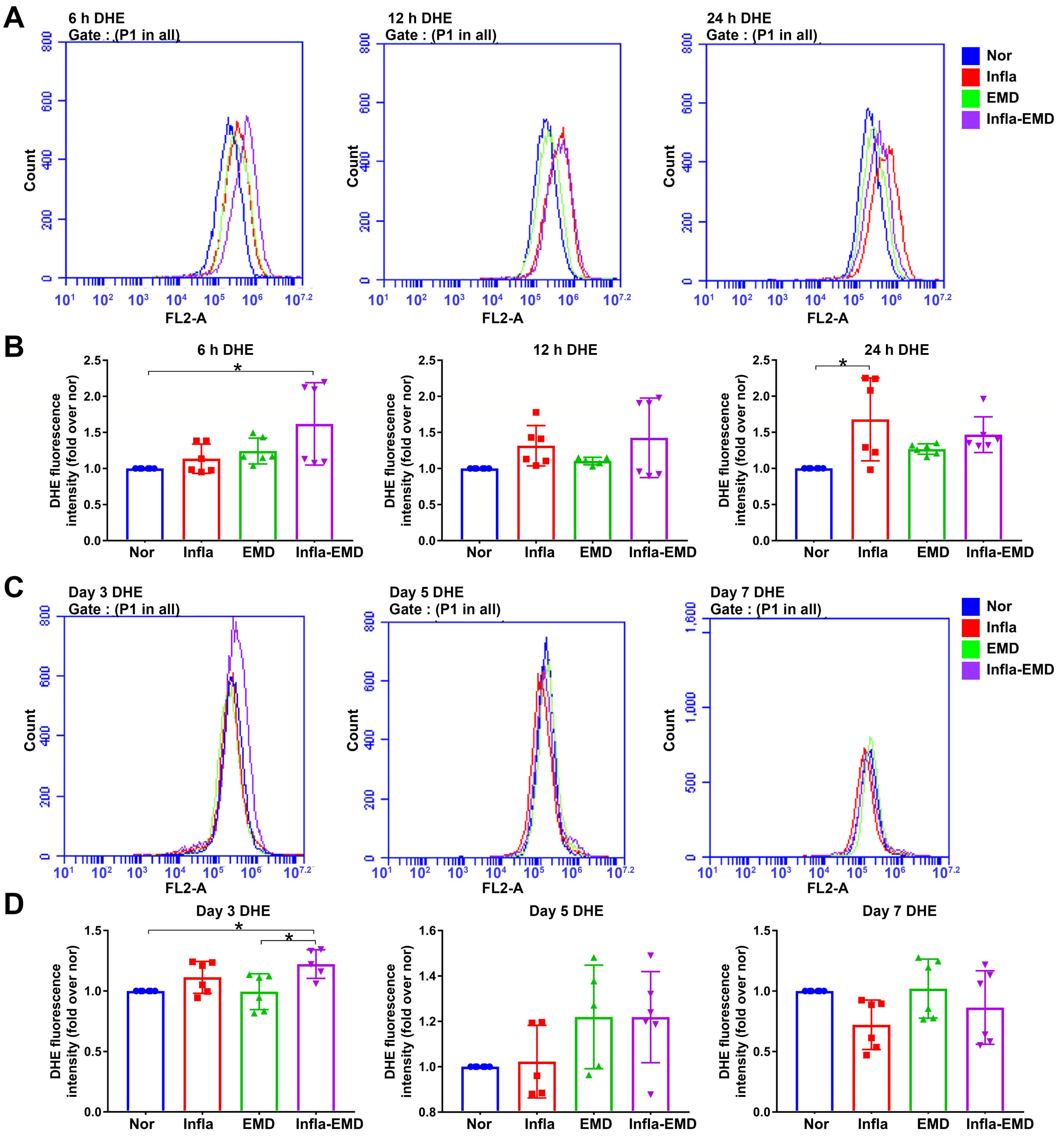

Fig. S3. A 3-day incubation was identified as the appropriate duration for detecting the intracellular ROS levels in PDLSCs. The cells were incubated in normal α-MEM medium (Nor), medium with the inflammatory cytokines TNF-α plus IL-1β (Infla), medium with the cementoblastic inducer EMD (EMD), or medium with both inflammatory cytokines and EMD (Infla-EMD). (**a**) Intracellular ROS levels in PDLSCs following incubation for 6, 12 and 24 h

determined with the aid of a DHE probe (flow cytometric analysis). (**b**) Quantification of the intracellular ROS levels (reflected by the relative fluorescence intensity of DHE). (**c**) Intracellular ROS levels in PDLSCs following incubation for 3, 5 and 7 days determined with the aid of a DHE probe (flow cytometric analysis). (**d**) Quantification of intracellular ROS levels (reflected by the relative fluorescence intensity of DHE). The data are shown as the means ± SDs for *n* from 5 to 6; $^*P < 0.05$ indicate significant differences between the four incubations at the same time point.

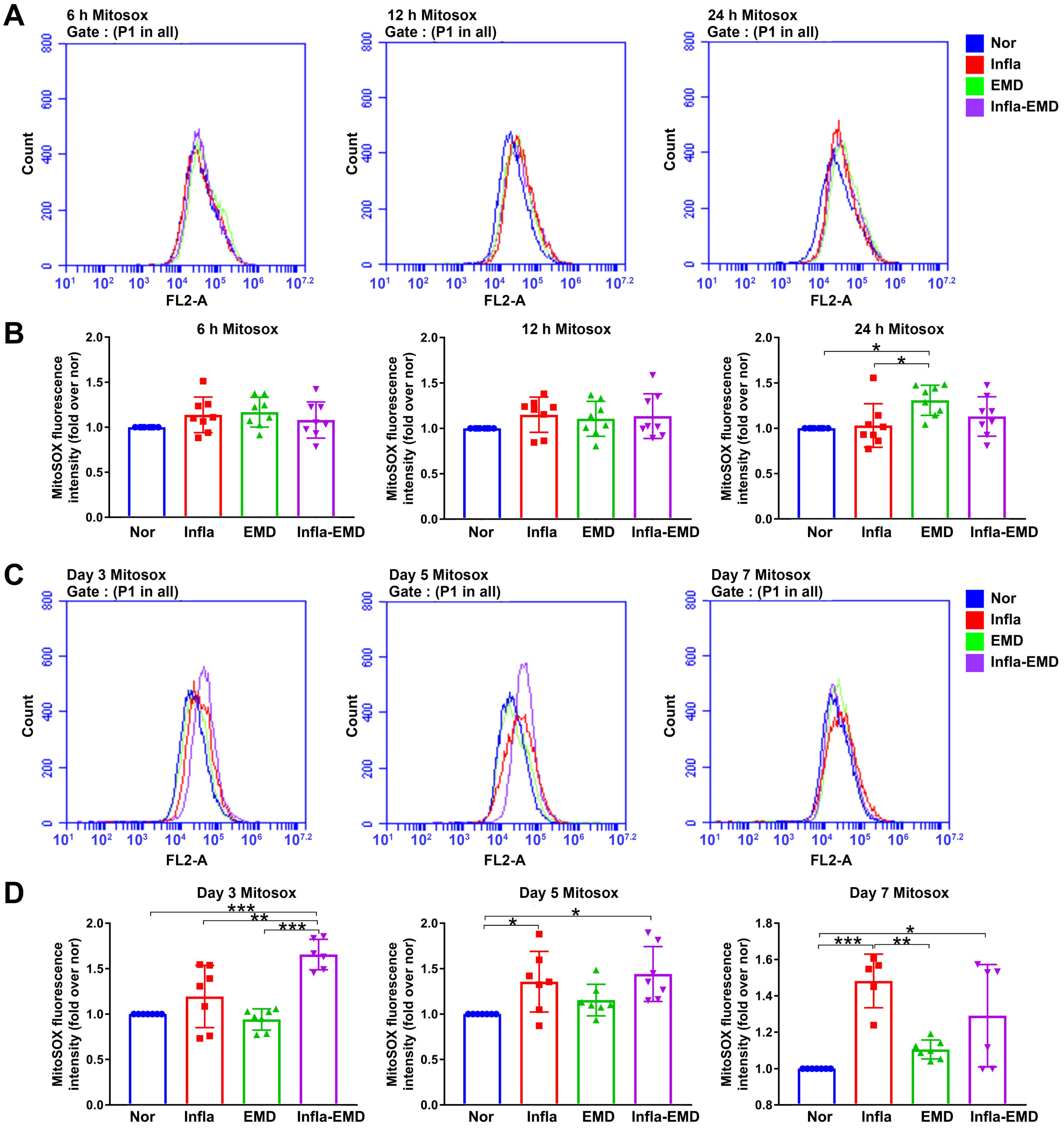

Fig. S4. A 3-day incubation was identified as the appropriate duration for detecting the mtROS levels in PDLSCs. The cells were incubated in normal α-MEM medium (Nor), medium with the inflammatory cytokines TNF-α plus IL-1β (Infla), medium with the cementoblastic inducer EMD (EMD), or medium with both inflammatory cytokines and EMD (Infla-EMD). (**a**) The mtROS levels in PDLSCs following incubation for 6, 12 and 24 h were determined with the aid

of a MitoSOX probe (flow cytometric analysis). (**b**) Quantification of mtROS levels (reflected by the relative fluorescence intensity of MitoSOX). (**c**) mtROS levels in PDLSCs following incubation for 3, 5 and 7 days determined with the aid of a MitoSOX probe (flow cytometric analysis). (**d**) Quantification of mtROS levels (reflected by the relative fluorescence intensity of MitoSOX). The data are shown as the means ± SDs for n from 5 to 8; *$P < 0.05$, **$P < 0.01$ and ***$P < 0.001$ indicate significant differences between the four incubations at the same time point.

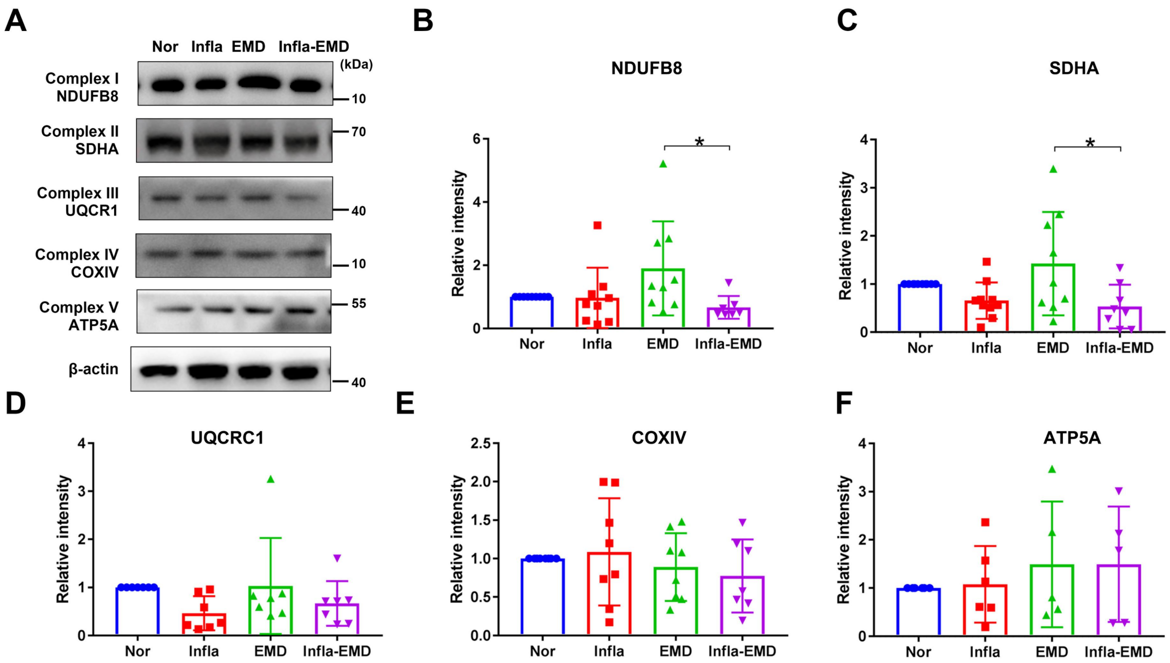

Fig. S5. Effects of inflammatory cytokines on mitochondrial complex-related protein expression. The cells were incubated in normal α-MEM medium (Nor), medium with the inflammatory cytokines TNF-α plus IL-1β (Infla), medium with the cementoblastic inducer EMD (EMD), or medium with both inflammatory cytokines and EMD (Infla-EMD). (**a**) Relative mitochondrial complex-related protein expression of NDUFB8 (subunit of complex I), SDHA (subunit of complex II), UQCRC1 (subunit of complex III), COXIV (subunit of complex IV) and ATP5A (subunit of complex V) determined by Western blot. (**b-f**) Semiquantitative analysis of NDUFB8 (**b**), SDHA (**c**), UQCRC1 (**d**), COXIV (**e**) and ATP5A (**f**) protein expression levels (normalized to β-actin) in terms of relative gray density. The data are shown as the means ± SDs for n from 5 to 10; *$P < 0.05$ indicate significant differences between the indicated columns.

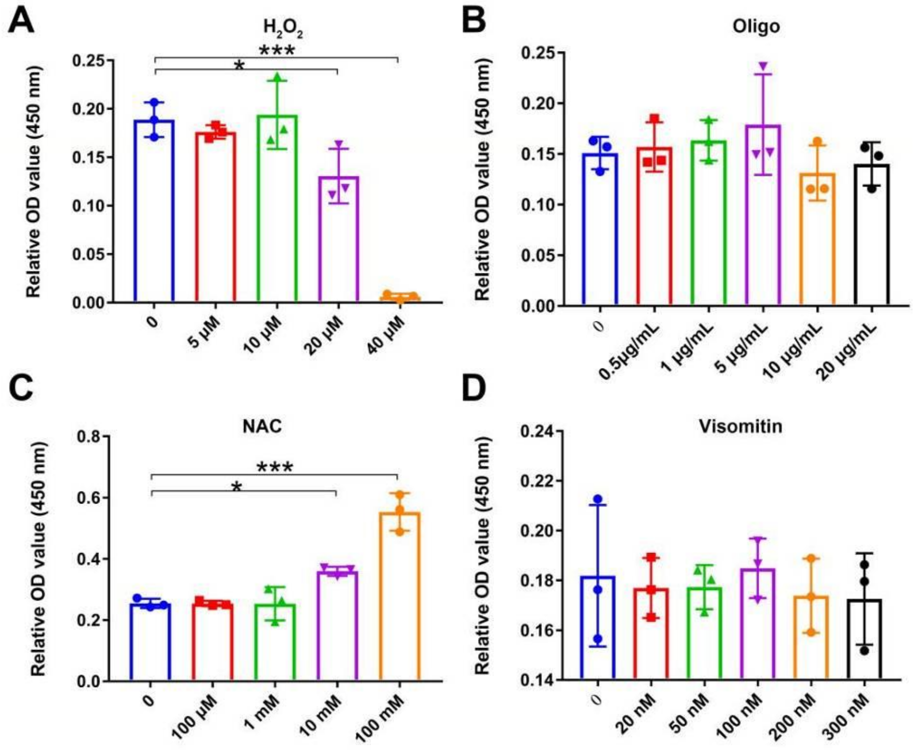

Fig. S6. Effects of various concentrations of H_2O_2, oligo, NAC and visomitin on the proliferation of PDLSCs. (**a**) Proliferation of PDLSCs following incubation with various concentrations of H_2O_2 determined by a CCK-8 assay. (**b**) Proliferation of PDLSCs following incubation with various concentrations of Oligo determined by a CCK-8 assay. (**c**) Proliferation of PDLSCs following incubation with various concentrations of NAC determined by a CCK-8 assay. (**d**) Proliferation of PDLSCs following incubation with various concentrations of Visomitin determined by a CCK-8 assay. The data are shown as the means ± SDs for n = 3; *P < 0.05 and ***P < 0.001 indicate significant differences between the indicated columns.

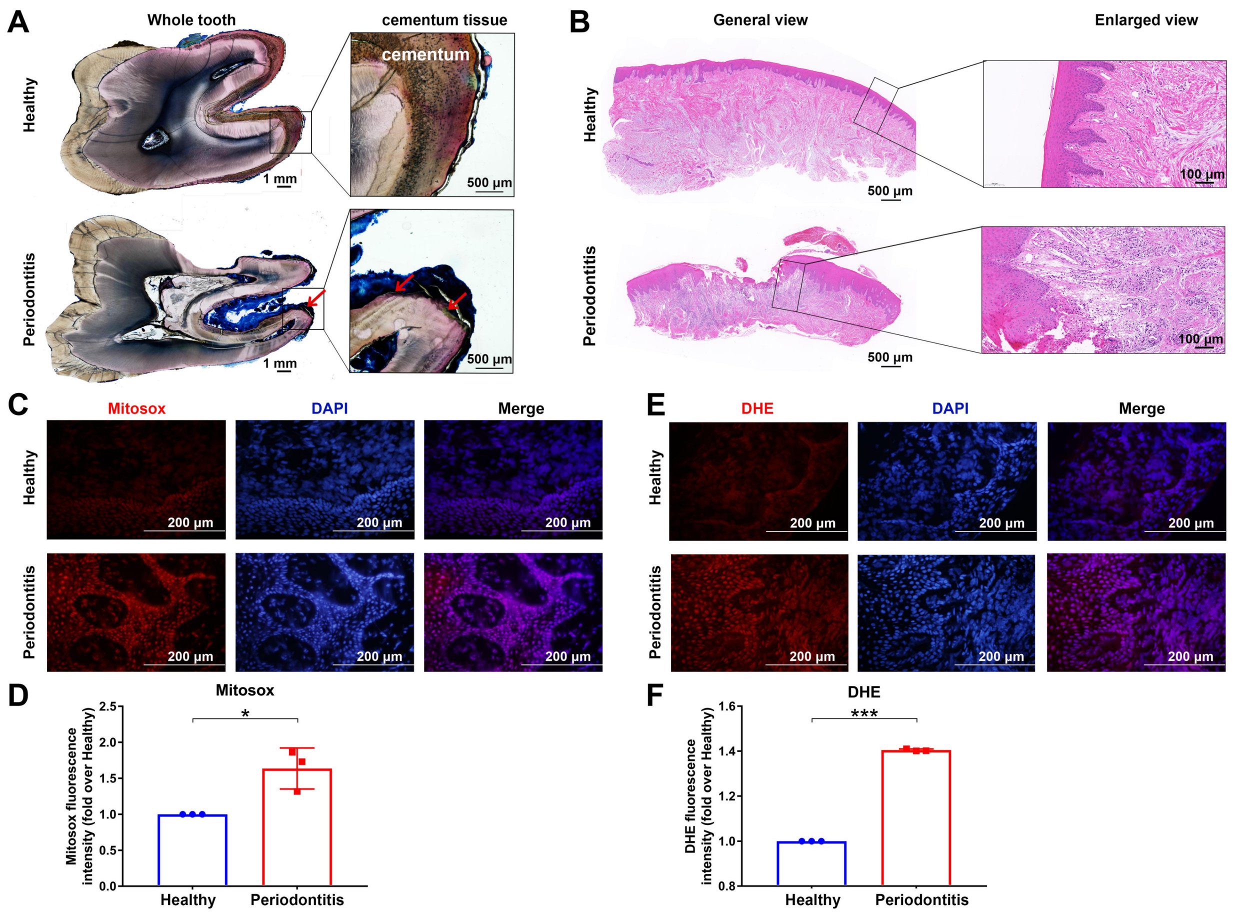

Fig. S7. Significantly higher mtROS and intracellular ROS levels were observed in gingival tissues derived from periodontally diseased teeth (periodontitis) than in gingival tissues of healthy teeth (healthy). (**a**) Histopathological assessment showing the healthy or damaged cementum of teeth in the healthy or periodontitis group, respectively (methylene blue-acid fusion staining; red arrows indicate cementum damage due to periodontitis; scale bar: 1 mm and 500 μm). (**b**) Histopathological assessment of gingival tissues derived from extracted teeth in the Healthy or Periodontitis group (H&E staining; scale bar: 500 μm and 100 μm). (**c**) mtROS expression in gingival tissues of the Healthy or Periodontitis group (MitoSOX staining; scale bar: 200 μm). (**d**) Qualification analysis of mtROS levels in gingival tissues of the Healthy or Periodontitis group. (**e**) Intracellular ROS expression in gingival tissues of the Healthy or Periodontitis group (DHE staining; scale bar: 200 μm). (**f**) Qualification analysis of the

intracellular ROS levels in gingival tissues of the Healthy or Periodontitis group. The data are shown as the means ± SDs for $n = 3$; *$P < 0.05$ and ***$P < 0.001$ indicate significant differences between the indicated columns.

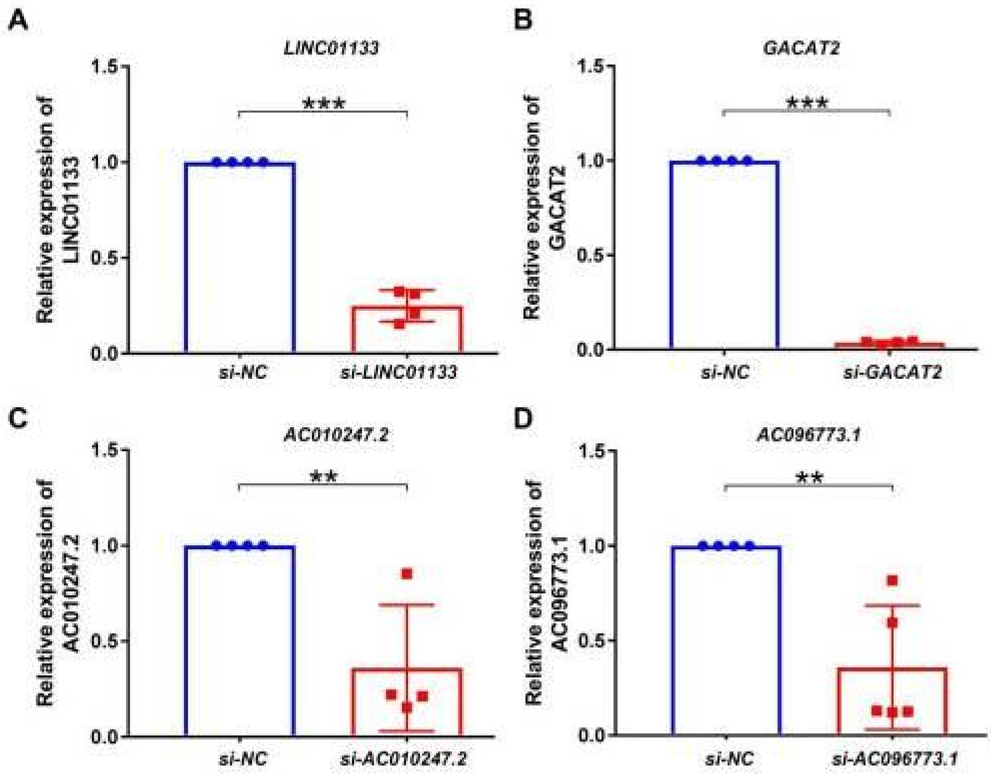

Fig. S8. Silencing efficiency of 4 lncRNAs (*AC010247.2, AC096773.1, LINC01133* and *GACAT2*) in PDLSCs transfected with specific siRNAs (qRT-PCR Assay). (**a**) Relative expression of *LINC01133* in PDLSCs transfected with si-NC and si-*LINC01133*. (**b**) Relative expression of *GACAT2* in PDLSCs transfected with si-NC and si-*GACAT2*. (**c**) Relative expression of *AC010247.2* in PDLSCs transfected with si-NC and si-*AC010247.2*. (**d**) Relative expression of *AC096773.1* in PDLSCs transfected with si-NC and si-*AC096773.1*. The data are shown as the means ± SDs for n from 4 to 5; $^{**}P < 0.01$ and $^{***}P < 0.001$ indicate significant differences between the indicated columns.

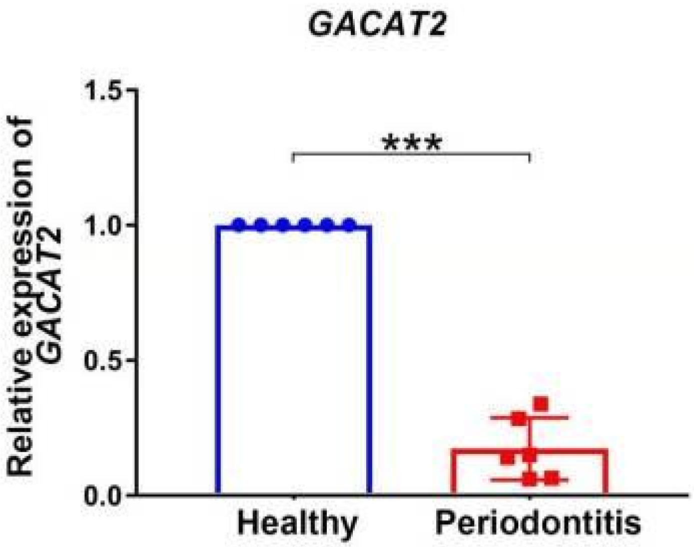

Fig. S9. *GACAT2* is downregulated in gingival tissues derived from periodontally diseased teeth (Periodontitis) compared with gingival tissues of healthy teeth (Healthy). The data are shown as the means ± SDs (n = 6); ***P < 0.001 indicate significant differences between the indicated columns.

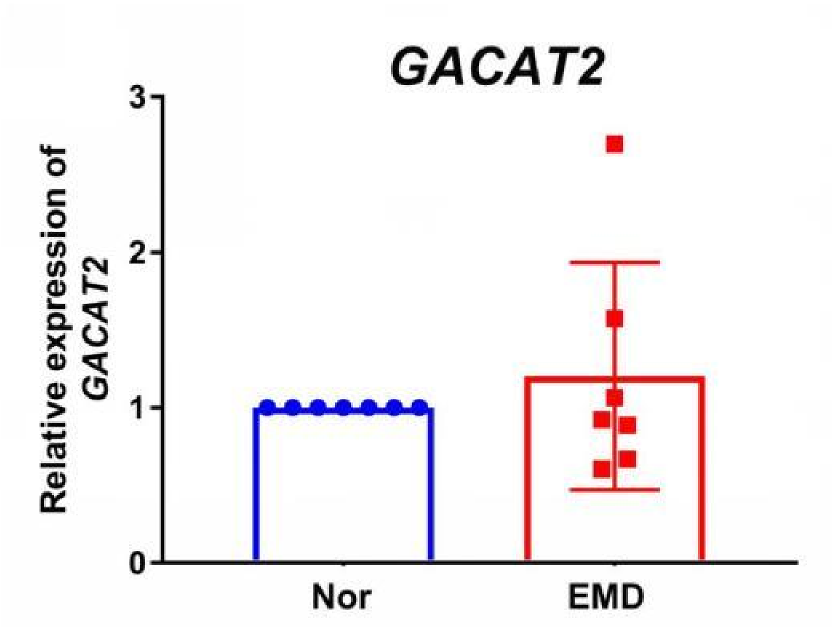

Fig. S10. No significant changes in the *GACAT2* expression levels were found between the PDLSCs from the Nor group and those from the EMD group. The data are shown as the means ± SDs (n = 7).

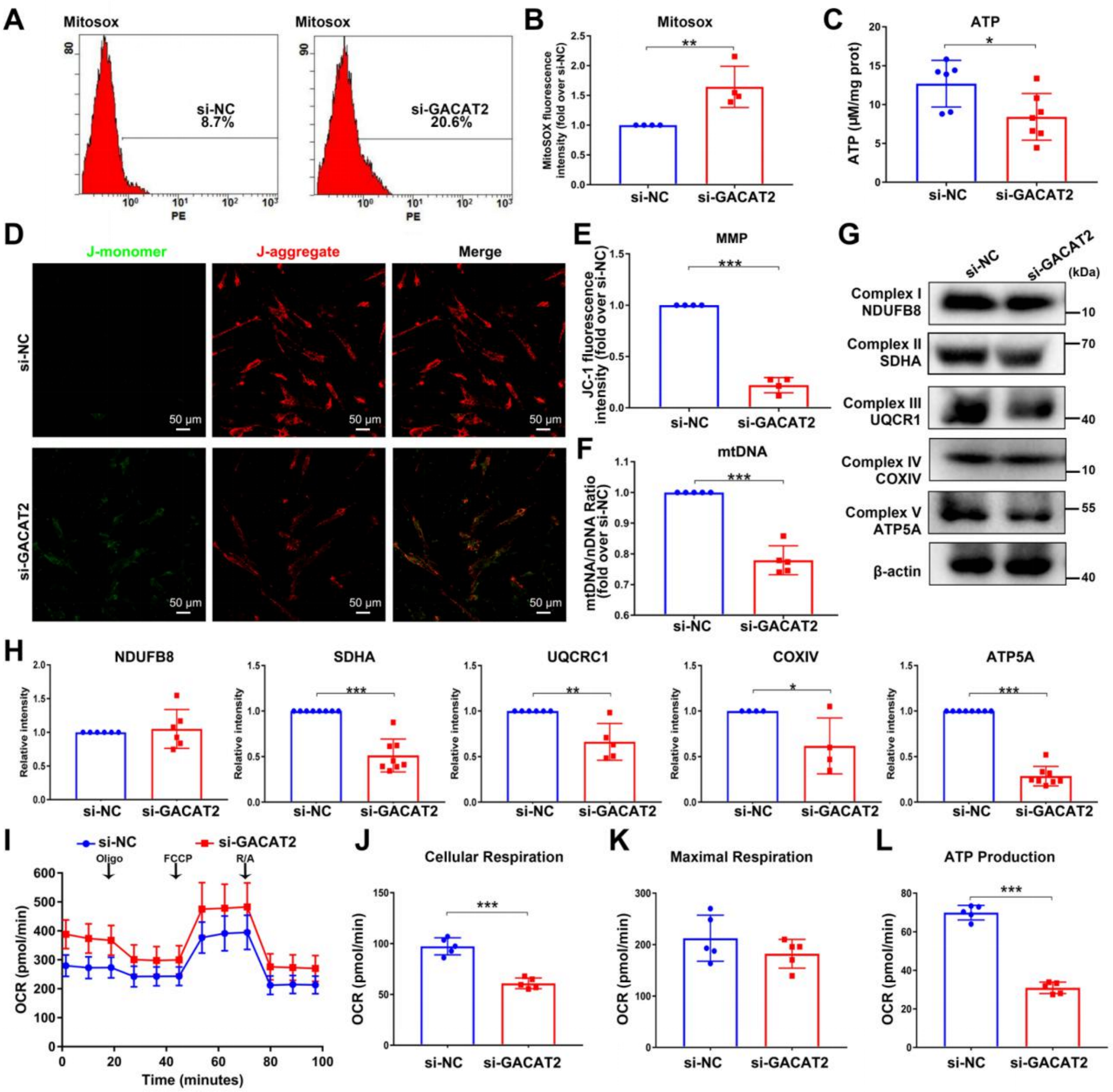

Fig. S11. Inhibition of *GACAT2* (si-*GACAT2*) causes mitochondrial dysfunction in PDLSCs. The cells were incubated in medium with EMD (a noninflammatory environment). (**a**) mtROS levels in PDLSCs determined with the aid of a MitoSOX probe (flow cytometric analysis). (**b**) Quantification of mtROS levels (reflected by the relative fluorescence intensity of MitoSOX). (**c**) Intracellular ATP contents (ATP assay). (**d**) The MMP was determined with the aid of a JC-1 probe (immunofluorescence staining; scale bar: 50 μm). (**e**) Quantification of MMP levels

(reflected by the relative ratio of red/green fluorescence intensity of JC-1). (**f**) mtDNA content determined by qRT-PCR. (**g**) Relative mitochondrial complex-related protein expression of NDUFB8 (subunit of complex I), SDHA (subunit of complex II), UQCRC1 (subunit of complex III), COXIV (subunit of complex IV) and ATP5A (subunit of complex V) determined by Western blot. (**h**) Semiquantitative analysis of protein expression levels (normalized to β-actin) in terms of relative gray density. (**i**) OCR of PDLSCs determined by a Seahorse Bioscience XF Analyzer; arrows indicate the sequential injection of 1 μM oligo, 1 μM FCCP and 2 μM R/A. (**j**) Quantification of cellular respiration (basic OCR value prior to Oligo injection). (**k**) Quantification of maximal respiration (differences between maximum rate measurement after FCCP injection and minimum rate measurement after R/A injection). (**l**) Quantification of ATP production (difference between final rate measurement prior to Oligo injection and minimum rate measurement after Oligo injection). The data are shown as the means ± SDs for n from 4 to 8; *$P < 0.05$, **$P < 0.01$ and ***$P < 0.001$ indicate significant differences between the indicated columns.

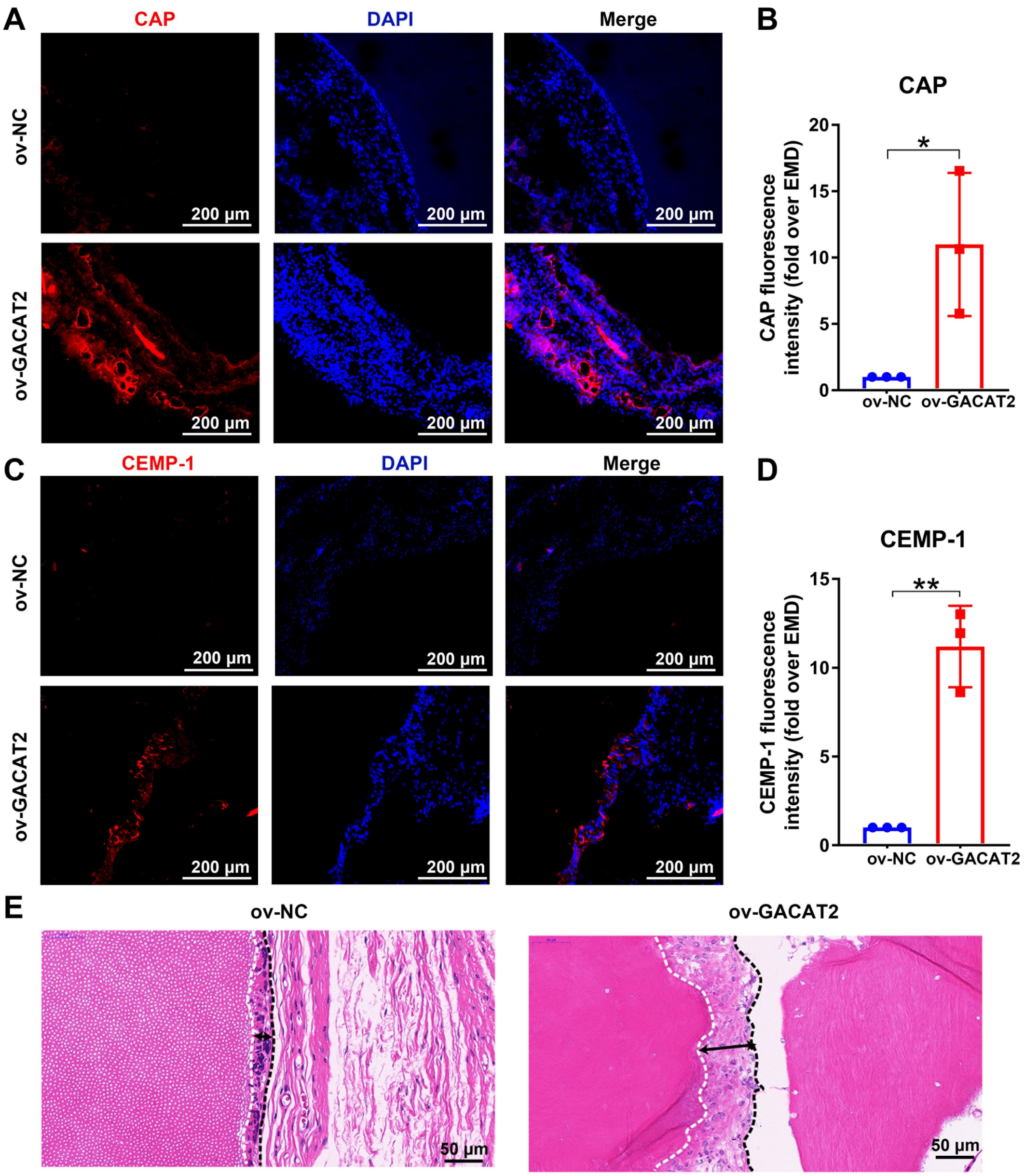

Fig. S12. Overexpression of *GACAT2* (ov-*GACAT2*) reverses inflammation-compromised cell cementogenesis in vivo. PDLSCs that were incubated in an inflammatory environment (EMD plus Vc and inflammatory cytokines) and transfected with ov-*GACAT2* (ov-*GACAT2*) or ov-NC (ov-NC) were subcutaneously transplanted into nude mice. (**a**) CAP expression in transplants derived from the ov-NC group or ov-*GACAT2* group (immunofluorescence staining; scale bar: 200 μm). (**b**) Qualification analysis of the CAP expression levels in transplants derived from the

ov-NC group or ov-*GACAT2* group. (**c**) CEMP-1 expression in transplants derived from the ov-NC group or ov-*GACAT2* group (immunofluorescence staining; scale bar: 200 μm). (**d**) Qualification analysis of the CEMP-1 expression levels in transplants derived from the ov-NC group or ov-*GACAT2* group. (**e**) Histopathological assessment of transplants derived from the ov-NC or ov-*GACAT2* group (H&E staining; arrows indicate cementum newly formed by PDLSC sheets; scale bar: 50 μm). The data are shown as the means ± SDs for $n = 3$; *$P < 0.05$ and **$P < 0.01$ indicate significant differences between the indicated columns.

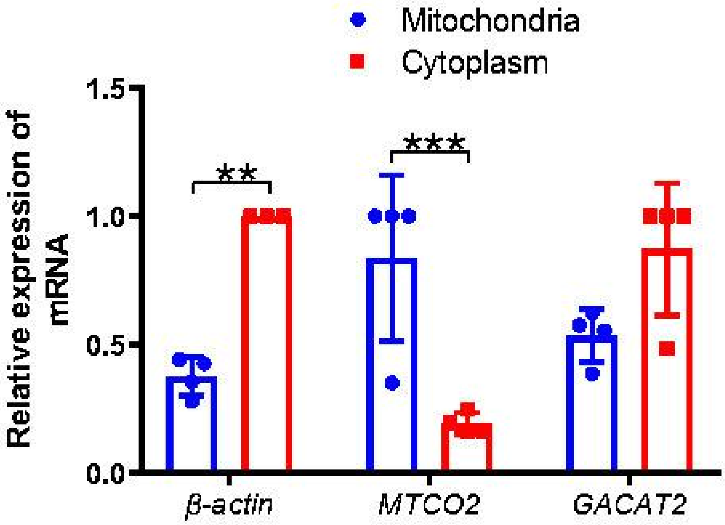

Fig. S13. Mitochondrial and cytoplasmic *GACAT2* were determined by mitochondrial fractionation analysis (*MTCO2* and *β-actin* served as the mitochondrial and cytoplasmic controls, respectively). The data are shown as the means ± SDs ($n = 4$); $^{**}P < 0.01$ and $^{***}P < 0.001$ indicate significant differences between the indicated columns.

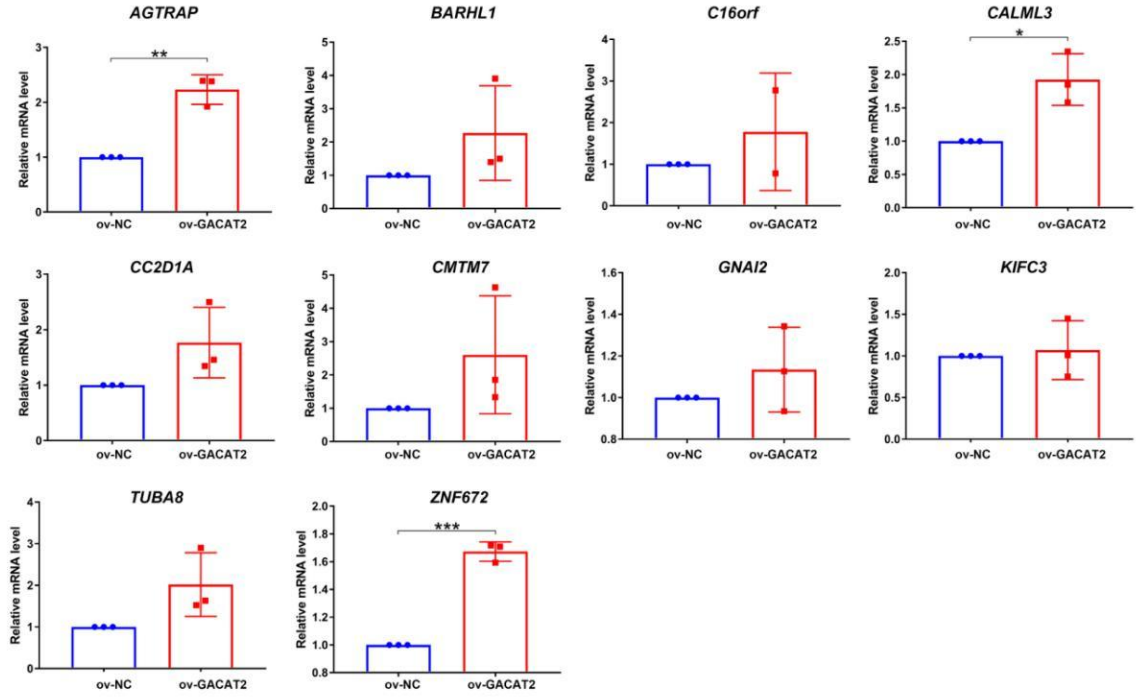

Fig. S14. Validation of potential mRNAs in the ceRNA network of *GACAT2* (qRT-PCR Assay). The data are shown as the means ± SDs ($n = 3$); $^*P < 0.05$, $^{**}P < 0.01$ and $^{***}P < 0.001$ indicate significant differences between the indicated columns.

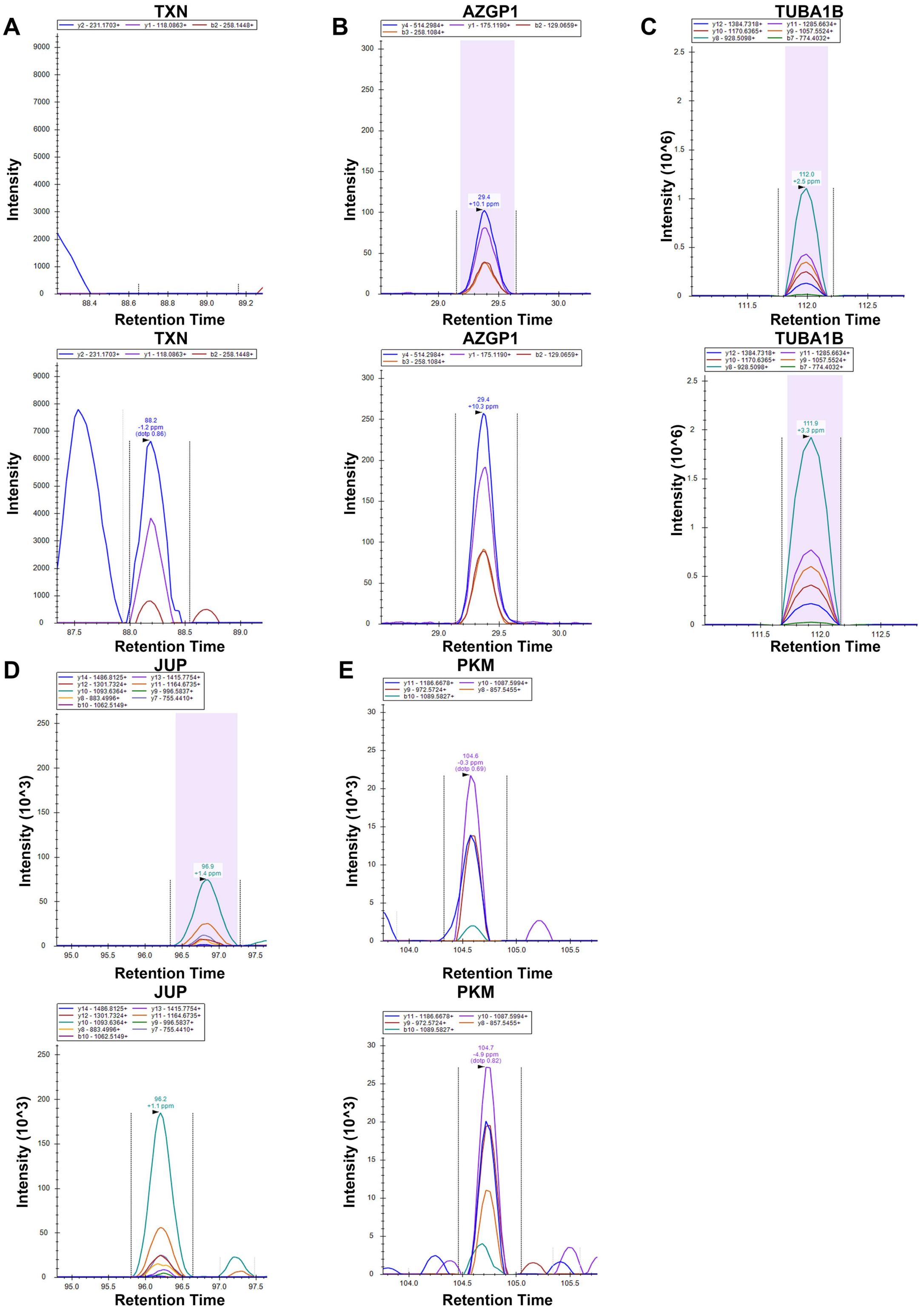

Fig. S15. Chromatograph of labeled peptides of potential GACAT2-binding proteins (TXN, AZGP1, TUBA1B, JUP and PKM1/2) (PRM assay). (**a**) Chromatograph of a labeled peptide

(EKLEATINELV) for the TXN protein verified by PRM assay. (**b**) Chromatograph of a labeled peptide (AGEVQEPELR) for the AZGP1 protein verified by PRM assay. (**c**) Chromatograph of a labeled peptide (AVFVDLEPTVIDEVR) for the protein TUBA1B verified by PRM assay. (**d**) Chromatograph of a labeled peptide (NLALCPANHAPLQEAAVIPR) for the JUP protein verified by PRM assay. (**e**) Chromatograph of a labeled peptide (IYVDDGLISLQVK) for the PKM1/2 protein verified by PRM assay.

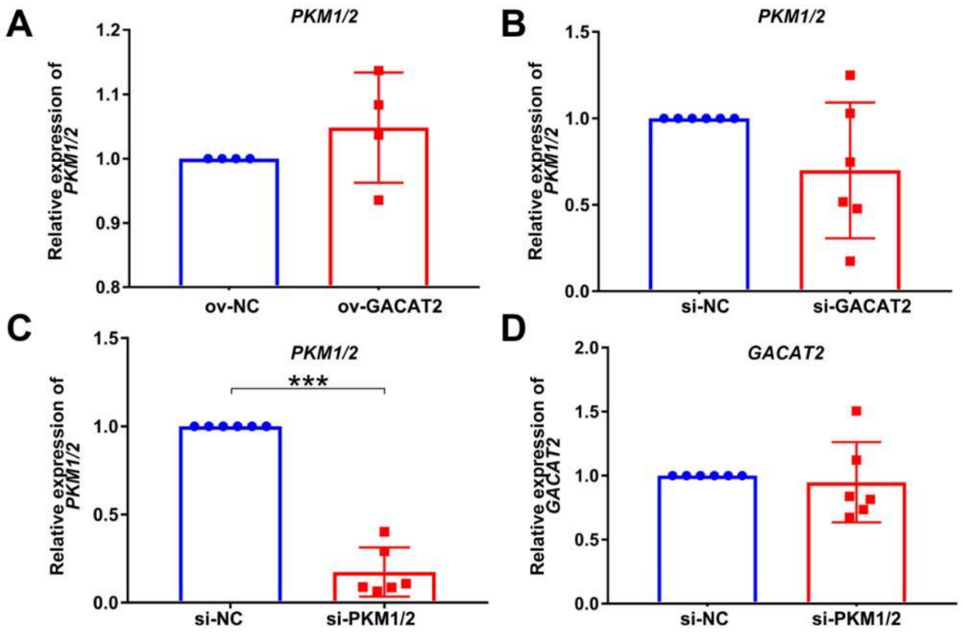

Fig. S16. *GACAT2* and *PKM1/2* do not affect each other at the gene level (qRT-PCR Assay). (**a**) Relative expression of *PKM1/2* in PDLSCs transfected with ov-NC or ov-*GACAT2* and incubated in medium with both inflammatory cytokines and EMD (inflammatory environment). (**b**) Relative gene expression levels of *PKM1/2* in PDLSCs transfected with si-NC or si-*GACAT2* and incubated in medium with the cementoblastic inducer EMD. (**c**) Silencing efficiency of si-*PKM1/2*. (**d**) Relative gene expression levels of *GACAT2* in PDLSCs transfected with si-NC or si-*PKM1/2* and incubated in medium with the cementoblastic inducer EMD. The data are shown as the means ± SDs for n from 4 to 6; ***$P < 0.001$ indicate significant differences between the indicated columns.

Supplementary Tables

Table S1. Information on the top 8 upregulated and top 8 downregulated lncRNAs in PDLSCs of the EMD group compared with those of the Infla-EMD group identified in the present study.

GeneSymbol	GeneID	Regulation	Fold Change	P-value	RNA length	Relationship	Source	Infla-EMD Raw Intensity	EMD Raw Intensity
G023182	G023182	down	20.4611742	0.00034716	3403	intronic antisense	RNA-seq	17.151887	290.099533
AC007938.3	ENSG00000270953	down	15.8593331	0.00046685	623	natural antisense	GENCODE	5	81.862403
LINC01638	ENSG00000233521	down	13.0436862	0.00064677	637	intergenic	GENCODE	15.623187	179.56207
AC017076.1	ENSG00000271947	down	10.5547163	0.00124715	578	intergenic	GENCODE	6.077737	59.197479
LINC01412	ENSG00000232606	down	10.0347486	0.00558546	362	intergenic	GENCODE	10.392902	97.042533
LINC01133	ENSG00000224259	down	9.4489213	0.00122793	1113	intergenic	GENCODE	80.852848	731.910063
LINC00856	ENSG00000230417	down	8.6529904	0.00012289	1249	exon sense-overlapping	CLSFL	63.316143	505.070657
GACAT2	ENSG00000265962	down	8.508416	0.00391955	818	natural antisense	GENCODE	132.121947	936.1817
PTGS2	ENSG00000073756	up	165.5024006	0.0003494	3690	exon sense-overlapping	GENCODE	8448.971433	52.203017
AC096773.1	ENSG00000250945	up	147.2913537	5.5999E-05	648	intergenic	GENCODE	910.7046	5.900829
AL390957.1	ENSG00000285280	up	96.5185631	1.2591E-05	435	intergenic	Refseq	2430.112667	23.184007
PDE10A	ENSG00000112541	up	49.3476006	0.04434491	1822	exon sense-overlapping	GENCODE	5567.211933	199.471638
G046815	G046815	up	47.5791909	7.0373E-05	1990	intergenic	RNA-seq:	300.96614	5.852916
XLOC_004067	XLOC_004067	up	45.9544999	5.1021E-05	601	intergenic	RNA-seq	246.826987	5
G051208	G051208	up	39.6385569	0.00273889	13722	intergenic	RNA-seq	271.382377	5.077367

AC010247.2	ENSG00000259436	up	35.6786733	0.00015909	1955	intronic antisense	GENCODE	2943.838033	79.669643

661 **Table S2.** Information on the ceRNA network of *GACAT2* in the present study.

Gene Symbol	CeNames	CeSymbols	CeTypes	Common Mirnas
GACAT2	ENST000003143 40	AGTRAP	protein_coding	hsa-miR-1207-5p,hsa-miR-122-5p,hsa-miR-1266-5p,hsa-miR-127-5p,hsa-miR-1538,hsa-miR-182-3p,hsa-miR-1910-5p,hsa-miR-34c-5p,hsa-miR-3652,hsa-miR-3918,hsa-miR-3928-5p,hsa-miR-4270,hsa-miR-4430,hsa-miR-4459,hsa-miR-4498,hsa-miR-4512,hsa-miR-4518,hsa-miR-4532,hsa-miR-4731-5p,hsa-miR-4745-3p,hsa-miR-4763-3p,hsa-miR-574-5p,hsa-miR-6132,hsa-miR-6165,hsa-miR-6741-5p,hsa-miR-6754-5p,hsa-miR-6805-5p,hsa-miR-6806-3p,hsa-miR-6836-5p,hsa-miR-6846-5p,hsa-miR-6848-5p,hsa-miR-762
GACAT2	ENST000003065 62	ZNF672	protein_coding	hsa-miR-1207-5p,hsa-miR-1292-5p,hsa-miR-1587,hsa-miR-218-2-3p,hsa-miR-23a-5p,hsa-miR-23b-5p,hsa-miR-34a-5p,hsa-miR-34c-5p,hsa-miR-3620-5p,hsa-miR-3652,hsa-miR-378g,hsa-miR-4270,hsa-miR-4430,hsa-miR-4459,hsa-miR-4492,hsa-miR-4498,hsa-miR-449a,hsa-miR-449b-5p,hsa-miR-4674,hsa-miR-4689,hsa-miR-4717-5p,hsa-miR-4722-5p,hsa-miR-4731-5p,hsa-miR-4763-3p,hsa-miR-5001-5p,hsa-miR-5006-5p,hsa-miR-637,hsa-miR-644a,hsa-miR-6858-5p,hsa-miR-6893-3p,hsa-miR-7108-5p,hsa-miR-7160-3p,hsa-miR-762,hsa-miR-875-3p,hsa-miR-939-3p
GACAT2	ENST000003136 01	GNAI2	protein_coding	hsa-miR-1207-5p,hsa-miR-125a-3p,hsa-miR-1266-5p,hsa-miR-1268a,hsa-miR-1268b,hsa-miR-1292-5p,hsa-miR-1587,hsa-miR-2861,hsa-miR-296-5p,hsa-miR-30a-5p,hsa-miR-30b-5p,hsa-miR-30c-5p,hsa-miR-30d-5p,hsa-miR-30e-5p,hsa-miR-3147,hsa-miR-3619-5p,hsa-miR-3620-5p,hsa-miR-3652,hsa-miR-3918,hsa-miR-4270,hsa-miR-4430,hsa-miR-4492,hsa-miR-4498,hsa-miR-4518,hsa-miR-4640-5p,hsa-miR-4722-5p,hsa-miR-4726-5p,hsa-miR-4731-5p,hsa-miR-4763-3p,hsa-miR-5001-5p,hsa-miR-5591-5p,hsa-miR-6132,hsa-miR-637,hsa-miR-6510-5p,hsa-miR-6515-5p,hsa-miR-663b,hsa-miR-6726-5p,hsa-miR-6754-5p,hsa-miR-6836-5p,hsa-miR-6846-5p,hsa-miR-6848-5p,hsa-miR-7160-3p,hsa-miR-762,hsa-miR-920
GACAT2	ENST000002993 20	C16orf71	protein_coding	hsa-miR-1182,hsa-miR-1183,hsa-miR-1207-5p,hsa-miR-1324,hsa-miR-1343-3p,hsa-miR-1538,hsa-miR-214-3p,hsa-miR-2861,hsa-miR-3607-3p,hsa-miR-3619-5p,hsa-miR-4492,hsa-miR-4498,hsa-miR-4512,hsa-miR-4745-3p,hsa-miR-4763-3p,hsa-miR-4779,hsa-miR-5001-5p,hsa-miR-5196-3p,hsa-miR-5693,hsa-miR-6132,hsa-miR-637,hsa-miR-6515-5p,hsa-miR-6783-3p,hsa-miR-6805-5p,hsa-miR-6836-5p,hsa-miR-6846-5p,hsa-miR-6848-5p,hsa-miR-761,hsa-miR-762,hsa-miR-7704
GACAT2	ENST000005412 40	KIFC3	protein_coding	hsa-miR-128-1-5p,hsa-miR-128-2-5p,hsa-miR-1538,hsa-miR-214-3p,hsa-miR-218-2-3p,hsa-miR-34a-5p,hsa-miR-34c-5p,hsa-miR-3619-5p,hsa-miR-4492,hsa-miR-4498,hsa-miR-4521,hsa-miR-4639-3p,hsa-miR-4689,hsa-miR-4721,hsa-miR-5001-5p,hsa-miR-5006-5p,hsa-miR-5693,hsa-miR-617,hsa-miR-6510-5p,hsa-miR-6776-5p,hsa-miR-6805-5p,hsa-miR-6846-5p,hsa-miR-6848-5p,hsa-miR-6858-5p,hsa-miR-7160-3p,hsa-miR-761,hsa-miR-762,hsa-miR-7703
GACAT2	ENST000003180 03	CC2D1A	protein_coding	hsa-miR-1538,hsa-miR-1587,hsa-miR-2861,hsa-miR-3620-5p,hsa-miR-378g,hsa-miR-3918,hsa-miR-4459,hsa-miR-4474-3p,hsa-miR-4492,hsa-miR-4498,hsa-miR-4640-5p,hsa-miR-4721,hsa-miR-4722-5p,hsa-miR-4726-5p,hsa-miR-4731-5p,hsa-miR-4745-3p,hsa-miR-4779,hsa-miR-5001-5p,hsa-miR-5693,hsa-miR-574-5p,hsa-miR-637,hsa-miR-644a,hsa-miR-663b,hsa-miR-6776-5p,hsa-miR-6846-5p,hsa-miR-6848-5p,hsa-miR-6893-3p,hsa-miR-7108-5p,hsa-miR-7160-3p,hsa-miR-762,hsa-miR-766-5p,hsa-miR-939-3p

GACAT2	ENST00000263610	BARHL1	protein_coding	hsa-miR-1207-5p,hsa-miR-1268a,hsa-miR-1268b,hsa-miR-1587,hsa-miR-2277-5p,hsa-miR-296-5p,hsa-miR-3127-3p,hsa-miR-3620-5p,hsa-miR-3656,hsa-miR-378g,hsa-miR-3918,hsa-miR-4283,hsa-miR-4492,hsa-miR-4498,hsa-miR-4707-5p,hsa-miR-4717-5p,hsa-miR-4731-5p,hsa-miR-4763-3p,hsa-miR-4767,hsa-miR-5001-5p,hsa-miR-5088-3p,hsa-miR-566,hsa-miR-6132,hsa-miR-637,hsa-miR-6720-3p,hsa-miR-6756-3p,hsa-miR-6836-5p,hsa-miR-6840-5p,hsa-miR-6846-5p,hsa-miR-6848-5p,hsa-miR-6854-5p,hsa-miR-7112-5p,hsa-miR-7113-5p,hsa-miR-7160-3p,hsa-miR-762,hsa-miR-7704,hsa-miR-920
GACAT2	ENST00000330423	TUBA8	protein_coding	hsa-miR-1207-5p,hsa-miR-1266-5p,hsa-miR-127-5p,hsa-miR-128-1-5p,hsa-miR-1343-3p,hsa-miR-1587,hsa-miR-214-3p,hsa-miR-2861,hsa-miR-3619-5p,hsa-miR-3620-5p,hsa-miR-378g,hsa-miR-3921,hsa-miR-3928-5p,hsa-miR-4270,hsa-miR-4430,hsa-miR-4492,hsa-miR-4498,hsa-miR-4512,hsa-miR-4640-5p,hsa-miR-4722-5p,hsa-miR-4726-5p,hsa-miR-4731-5p,hsa-miR-4763-3p,hsa-miR-5001-5p,hsa-miR-5693,hsa-miR-663b,hsa-miR-6754-5p,hsa-miR-6776-5p,hsa-miR-6806-3p,hsa-miR-6854-5p,hsa-miR-7113-5p,hsa-miR-762
GACAT2	ENST00000315238	CALML3	protein_coding	hsa-miR-1207-5p,hsa-miR-1343-3p,hsa-miR-1587,hsa-miR-2277-5p,hsa-miR-3180-5p,hsa-miR-34a-5p,hsa-miR-34c-5p,hsa-miR-3620-5p,hsa-miR-4459,hsa-miR-449a,hsa-miR-449b-5p,hsa-miR-4639-3p,hsa-miR-4721,hsa-miR-4722-5p,hsa-miR-4731-5p,hsa-miR-4734,hsa-miR-4763-3p,hsa-miR-5001-5p,hsa-miR-5591-5p,hsa-miR-6515-5p,hsa-miR-665,hsa-miR-6726-5p,hsa-miR-6741-5p,hsa-miR-6776-5p,hsa-miR-6846-5p,hsa-miR-6848-5p,hsa-miR-7108-5p,hsa-miR-762,hsa-miR-766-5p,hsa-miR-920,hsa-miR-939-3p
GACAT2	ENST00000334983	CMTM7	protein_coding	hsa-miR-1180-3p,hsa-miR-1266-5p,hsa-miR-1287-3p,hsa-miR-1324,hsa-miR-1343-3p,hsa-miR-182-3p,hsa-miR-2861,hsa-miR-30a-5p,hsa-miR-30b-5p,hsa-miR-30c-5p,hsa-miR-30d-5p,hsa-miR-30e-5p,hsa-miR-3127-3p,hsa-miR-3160-5p,hsa-miR-3619-5p,hsa-miR-3652,hsa-miR-4270,hsa-miR-4430,hsa-miR-4459,hsa-miR-4492,hsa-miR-4498,hsa-miR-4518,hsa-miR-4640-5p,hsa-miR-4689,hsa-miR-4722-5p,hsa-miR-4726-5p,hsa-miR-4768-3p,hsa-miR-496,hsa-miR-5001-5p,hsa-miR-548q,hsa-miR-5591-5p,hsa-miR-6165,hsa-miR-6510-5p,hsa-miR-6515-5p,hsa-miR-6726-5p,hsa-miR-6754-5p,hsa-miR-6756-3p,hsa-miR-6783-3p,hsa-miR-6817-3p,hsa-miR-6854-5p,hsa-miR-6858-5p,hsa-miR-762,hsa-miR-766-5p,hsa-miR-920

Table S3. Information on 15 *GACAT2*-binding proteins screened by ChIRP-MS assay in the present study.

Gene Name	Unique. peptides	MS.MS. count	MS.MS. count.CON	MS.MS. count.LNC	MS.MS. count.U1	LNC	U1	Ctrl	fc..LNC--Ctrl
AZGP1	2	4	0	2	2	2	2	0	2.072698
CASP14	4	6	0	1	5	1	5	0	1.380376
CALM2	2	1	0	1	0	1	0	0	1.380376
KPRP	12	62	10	14	38	14	38	10	1.091905
RTN4	2	3	1	2	0	2	0	1	1.072698
KRT2	30	549	144	137	268	137	268	144	0.605777
FLG2	4	14	3	3	8	3	8	3	0.53856
GAPDH	4	31	10	9	12	9	12	10	0.48826
SLC3A2	2	2	1	1	0	1	0	1	0.380376
TXN	2	4	1	1	2	1	2	1	0.380376
KRT10	24	743	237	192	314	192	314	237	0.375925
PKM	12	23	11	9	3	9	3	11	0.362729
TUBA1B	11	32	14	11	7	11	7	14	0.313207
JUP	11	18	5	4	9	4	9	5	0.305183
DCD	5	16	5	4	7	4	7	5	0.305183

Table S4. Quantification of labeled reference peptides for *GACAT2*-binding proteins determined by PRM assay in the present study.

Protein Name	Gene Name	LNC	CON	Fold change (LNC/CON)
sp|P10599|THIO_HUMAN	TXN	50855	0	INF
sp|P25311|ZA2G_HUMAN	AZGP1	1753000	709760	2.469848963
sp|P68363|TBA1B_HUMAN	TUBA1B	10858000	4444300	2.443129402
sp|P14923|PLAK_HUMAN	JUP	702570	305250	2.301621622
sp|P14618|KPYM_HUMAN	PKM	176360	109850	1.605461994
tr|E7EUT5|E7EUT5_HUMAN	GAPDH	7673500	5193600	1.477491528
sp|P31944|CASPE_HUMAN	CASP14	302880	237490	1.275337909
sp|Q5D862|FILA2_HUMAN	FLG2	6885500	5907900	1.165473349
sp|Q5T749|KPRP_HUMAN	KPRP	420810	455020	0.924816492
sp|P81605|DCD_HUMAN	DCD	121130	141200	0.85786119
sp|P35908|K22E_HUMAN	KRT2	18491000	23763000	0.77814249
sp|P13645|K1C10_HUMAN	KRT10	1829800	2568900	0.712289307
tr|F8W914|F8W914_HUMAN	RTN4	88031	153350	0.57405282
tr|F5GZS6|F5GZS6_HUMAN	SLC3A2	99200	185310	0.535319195
